# Processing Technologies for Bee Products: An Overview of Recent Developments and Perspectives

**DOI:** 10.3389/fnut.2021.727181

**Published:** 2021-11-03

**Authors:** Xuan Luo, Yating Dong, Chen Gu, Xueli Zhang, Haile Ma

**Affiliations:** School of Food and Biological Engineering, Jiangsu University, Zhenjiang, China

**Keywords:** bee products, processing techniques, drying, wall-breaking, extraction, identification, quality control

## Abstract

Increased demand for a more balanced, healthy, and safe diet has accelerated studies on natural bee products (including honey, bee bread, bee collected pollen royal jelly, propolis, beeswax, and bee venom) over the past decade. Advanced food processing techniques, such as ultrasonication and microwave and infrared (IR) irradiation, either has gained popularity as alternatives or combined with conventional processing techniques for diverse applications in apiculture products at laboratory or industrial scale. The processing techniques used for each bee products have comprehensively summarized in this review, including drying (traditional drying, infrared drying, microwave-assisted traditional drying or vacuum drying, and low temperature high velocity-assisted fluidized bed drying), storage, extraction, isolation, and identification; the assessment methods related to the quality control of bee products are also fully mentioned. The different processing techniques applied in bee products aim to provide more healthy active ingredients largely and effectively. Furthermore, improved the product quality with a shorter processing time and reduced operational cost are achieved using conventional or emerging processing techniques. This review will increase the positive ratings of the combined new processing techniques according to the needs of the bee products. The importance of the models for process optimization on a large scale is also emphasized in the future.

## Introduction

Natural products and preparations for food and nutritional supplement or dietetic purposes have been used in folk medicine for several years (1). Apiculture products have long been used for phytotherapy and diet because of their positive effects on health (2, 3).

The history of bee products and their therapeutic effects date back to the ancient times. Healing properties of the bee products are recorded in many religious texts, including the Bible, Vedas, and Quran (4, 5). Bee collected pollen (BCP) and bee bread (BB) are used as food supplements for humans (6), even the Greeks believed that BCP and honey were the food of kings, which can give them the youthfulness and life. Nowadays, bee products [honey, royal jelly (RJ), propolis, beeswax, bee venom (BV), bee collected pollen (BCP), and bee bread (BB)] as natural medicines are gaining prominence for the full of bioactive compounds that are associated with their high bioactive molecule content (7), beneficial health properties (4, 8), including powerful healing properties. Thus, there is an increasing demand for these natural bee products currently.

The biologically active components of bee products include carbohydrates (9), proteins, peptides (10), lipids (11), vitamins (12, 13), minerals (14), polyphenols, flavonoids (15), terpenoids (16, 17), and a small amount of other compounds. The nutritional value of their chemical components certainly improves health and body functioning (12). Multiple studies also shown that for the active compounds contained in the bee products made them possess excellent antioxidant (17), antimicrobial (18, 19), anti-inflammatory, immunomodulatory (20), antiproliferative (21), anticarcinogenic, antitumor (22), and antiallergic properties. Served in the form of tablets, capsules, powders, granules, candy bars, oral liquids, and tonics or as received, bee products are particularly recommended as dietary supplements for human consumption (14, 23).

China is a large global producer of apiculture commodities, to fully develop these resources, maintain quality at the desired level, achieve maximum benefits and nutritional values, and produce each type of bee product as a dietary supplement for human beings, more effective processing techniques are badly needed to produce more nutritional and high-quality apiculture products.

Many external factors, including, temperature, humidity affected the physicochemical properties of bee products, even the processing treatments, such as ultrasonic assisted extraction, microwave-assisted drying (MWD), and analytical techniques (24, 25) can strongly influence the content of the active ingredients and their bioactivity. An integrated approach would be useful and efficient for the preservation of bioactivity ingredients during the production and processing stages. Physical processing of bee products focus on changing the moisture content (26), enhancing the extraction (27, 28), modifying the external structures (29).

In previous, several reviews have focused on the bioactivity and therapeutic properties (18, 30–33) of bee products; bee products active ingredients from different botanical origins and regions (34, 35); extraction (11) and analytical techniques (36) have been used to obtain, purify, identify, characterize, and/or quantify bioactive compounds (37). However, few reviews have comprehensively summarized the processing technologies applied in the bee products.

Thus, this review aims to present recent developments in processing techniques applied in bee products, which will strengthen the existing knowledge regarding bee product processing techniques and proposing potential combine processing strategies for better processing the bee products.

## Bee Products

**Figure 1:**
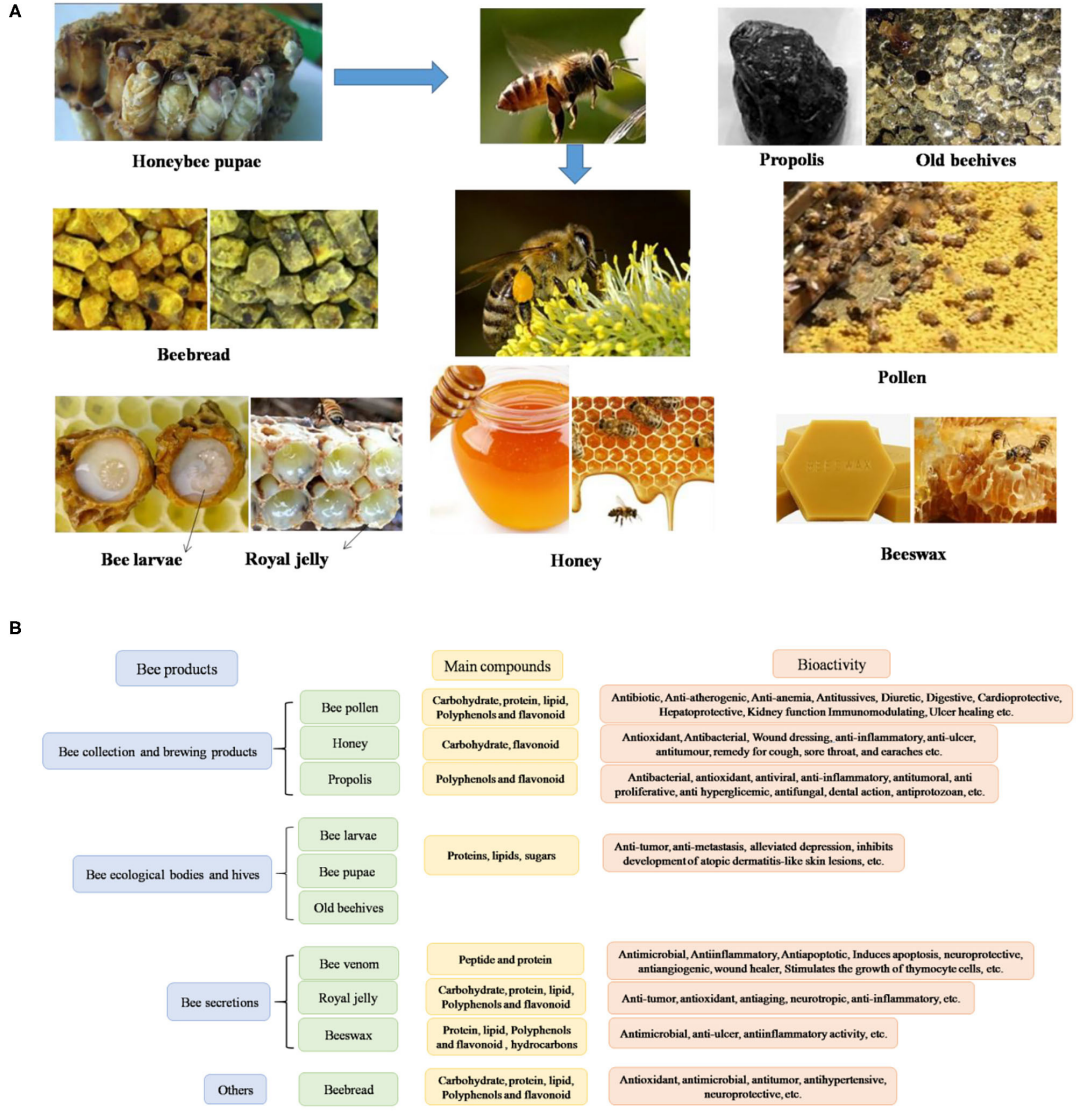
Bee products **(A)** and their bioactivities **(B)**. Cite from Pasupuleti et al. (40).

**Figure 2:**
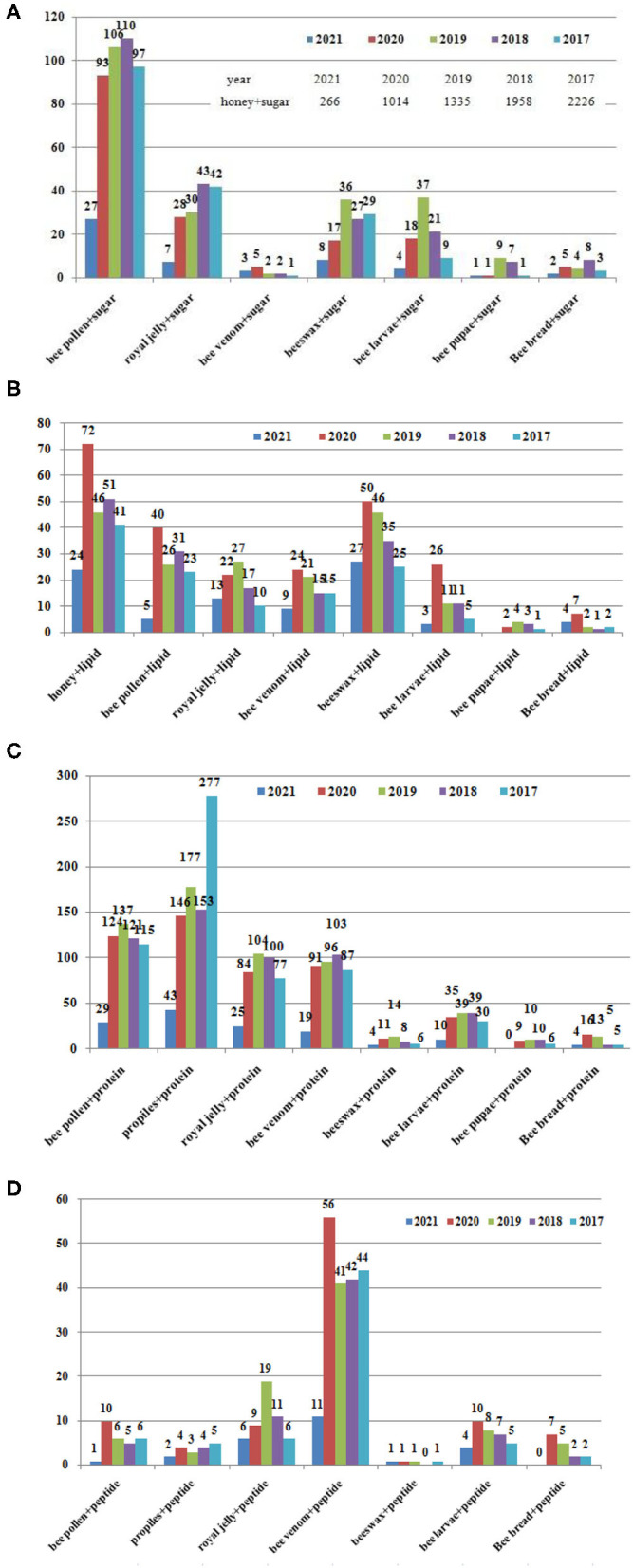
Papers related to bee products published in the recent 5 years (2017–2021). Data from the Web of Science on 4.25. **(A)** Sugar, **(B)** lipid, **(C)** protein, and **(D)** peptide.

Honeybee-derived products are used as traditional complementary medicines worldwide, especially in oriental countries (38). Bee products can be divided into three categories: (1) bee collection and brewing products, such as propolis, honey and BCP, BB; (2) Bee secretions, such as RJ, beeswax, and BV; and (3) bee ecological bodies and hives, such as bee larvae, bee corpses, and old beehives (39) (Figure 1). Hive products and their apitherapy have a long history dates back to the ancient times, which have been used in phytotherapy and diet for their powerful healing properties (6). Over the past 5 years, several research papers have been published focus on the beneficial compounds, data from the web of science were shown in Figure 2.

### Bee Collection and Brewing Products

#### Honey

Reports of ancient populations using honey for both nutritional and medicinal properties can be traced back to nearly 5,500 years ago (41, 42). Honey as a natural product produced from the nectar of flowers by honeybees (43). Because of its high nutritive components, it has been traditionally used to treat wounds and diseases of the gut, including gastric ulcers, used as a remedy for sore throat, cough, hepatitis, thirst, earaches, hiccups, fatigue, worm infections, tuberculosis, dizziness, constipation, piles, eczema (44–46).

Around 300 types of honey have been identified (47). The main component of honey is carbohydrate, which contributes 95–97% of its dry weight (48), predominantly fructose and glucose (40, 49, 50). Furthermore, honey contains proteins (0.5%), vitamins, more than 20 amino acids, minerals (0.04–0.2%), organic acids (0.57%), enzymes (sucrose, diastase or amylase; CAT; invertase, α-glucosidase; and glucose oxidase), and solid particles derived from the honey collection (51). Flavonoids and polyphenols are the two main bioactive (antioxidants) molecules present in honey (41, 52, 53).

#### Bee Pollen and Bee Bread

Both BCP and BB are rich in nutritional components like sugars, proteins, fatty acids (including ω-3 and ω-6 fatty acids), essential amino acids, vitamins, and even macro-and microelements (54).

Global production of BCP is ~1,500 tons per year. The largest producers are China, Australia, and Argentina (55). As a well-known functional food, the main primary constituents of the BCP are carbohydrates (12, 56, 57), and water, which are rich in protein (58, 59), lipids (57, 60–62), dietary fiber, and mineral contents, as well as vitamins and antioxidants (63).

BCP is the basic food for worker bees for RJ production or directly as food (larval queen and worker larvae) because of the large amount of proteins and lipids in it (64–66). The use of BCP for human nutrition on a larger scale began only after the Second World War (67, 68).

BB is a kind of hive product of lactic acid fermentation of BCP, which is unexplored by many beekeepers (52).

BCP are partly destroyed after fermentation, higher nutritional value and bioavailability it possess make it better digestibility than BCP (69). BB contains a few proteins and fats but an abundance of easily assimilated carbohydrates, free amino acids, and lactic acid. The conversion of BCP to BB is a result of biochemical changes, which are mediated by microbial metabolism, principal lactic acid fermentation by bacteria and yeasts (33).

#### Propolis

Propolis (bee glue) is a sticky resinous material that released from bud exudates, flowers, and leaves modified by bee secretions (enzymes) and wax (70, 71), which are present in bee hives and used by honeybees as a cementing material to close open spaces and cracks occurring in their hives (72). Propolis has been utilized as a helpful drug and nourishment for overall well-being since 300 BCE (73, 74). In 1908, the first scientific work was published about the propolis chemical properties and composition (73).

The composition of propolis varies depending on the geographical, vegetation, and ecological region, and even the time and method of the collection (75, 76). In general, propolis is composed of 45–55% vegetable balms and resins (flavonoids, phenolic acids, and esters), 8–35% wax, 5–10% essential oils and aromatics (including pinene, viridiflorol, eudesmol, and tricosane), 5% pollen, 5% fatty acids, and 5% other organic compounds, and minerals (77). Flavonoids, aromatic acids, chlorogenic acids, diterpenic acids, phenolic compounds and their derivatives, fatty acids, prenylated benzophenones, prenylated phenylpropanoid acids, and other secondary metabolites appear to be the principal components responsible for the biological activities of propolis (21, 78).

### Bee Secretions

#### RJ

RJ is produced by nurse bees to feed young worker larvae during the first 3 days and the entire life of queen bees (79–81). RJ is known as a “superfood,” and considered as one of the most appreciated and valued natural products that has been primarily used in traditional medicines, health food, and cosmetics (82). There is a long history about the RJ production techniques. In 1921, Sherlock Holmes simply use a syringe to suck the RJ out of a naturally queen cell. By the 1950 s, the small-scale production and sale of RJ was started by the Mexican, French, and Italian beekeepers (83). China began producing RJ using the method reported in a French literature in 1957 (84).

China is the largest producer and exporter of RJ worldwide, with more than 4,000 tons production annually 90% of the total RJ production are exported to Japan, Europe, and the United States and more than a $2.5 billion market (84, 85). Fresh RJ contains (60–70% w/w) water. The dry matter of the RJ is composed of protein (27–41%), carbohydrates (~30%), lipids (8–19%), minerals, some vitamins, and trace elements (13, 58, 86–88).

#### Beeswax

Beeswax is a natural biological polymer (89) isolated from bee secretions, and its chemical composition varies from geographical zones and bee species, although its concentrations may vary depending on the honeybee species and geographical origin. In 150 BC, beeswax was one of the components in cosmetic cream created by ancient physician (90), and it also played an important role in ancient and traditional Indian medicine (91). Beeswax are primarily composed of esters, fatty acids, alcohols and hydrocarbons, linear wax monoesters, free fatty alcohols, hydroxy monoesters derived from palmitic, oleic acids, 15-hydroxypalmitic, and complex wax esters containing 15-hydroxypalmitic acid and diols (92). Beeswax are multicomponent solid mixture at room temperature (93), and used as an additive in the food industry, cosmetics, and candles (16). Beeswax is a natural biological polymer (94), which is used as a thickener, drug carrier, binder, and a release retardant in pharmaceutical preparations (95).

#### BV

BV is a discovery dates back to ancient Egypt more than 6,000 years (96). As a therapeutic modality, BV has been used since the second century BC in Eastern Asia (97). BV is a complex natural mixture produced by *A. mellifera*, an European honeybee (98, 99). It has been widely used as a traditional medicine for treating various diseases (8). It is known to contain peptides [mast cell degranulating (MCD) peptide, melittin, apamin, and adolapin]; enzymes (phospholipase A2 (PLA2) and hyaluronidase); amino acids and volatile compounds (10, 100).

### Bee Ecological Bodies and Hives

#### Bee Larvae and Pupae

The life cycle of the honeybee has four major stages: egg, larva, pupa, and adult. The larval stage usually lasts ~6 days (101). As early as 1200 BC, the ancient book “Erya” recorded the eating of bees. The silk book “Fifty-two Cases” an ancient medical prescription unearthed in Mawangdui, Changsha, contains a prescription for treating diseases with bee embryos and bees (39). Honeybee larva was first recorded in the earliest extant classic on pharmacology for traditional Chinese medicine, “Shen nong ben cao jing” (102). In China, honeybee larvae have been used to treat and prevent various diseases for more than 1,000 years. Previous study reported that lyophilized powder of honeybee larvae could alleviate tinnitus-associated depression (103).

The protein and fat content of *A. mellifera* (larvae and pupae) were 42 ± 0.4–50 ± 1.6 and 19 ± 1.8–21 ± 1.5, respectively, and the minerals were 3 ± 1.3–4 ± 1.6 (104). As the larvae progressed to the imago stage, carbohydrate and fat contents decreased from 46.1 and 14.5% to 30.6 and 6.9%, whereas protein content increased from 35.3 to 51%. Ramos-Elorduy reported the protein content for pupae and bee larvae were 42 and 49% (dry weight), respectively (105). The pupae and larvae of the honeybee become an especially good source of essential amino acids [16.45/100 g of honey bee larvae powder (HLP)] and fatty acids (106). In addition, honey bee larvae powder extract (HLE) is full of total phenolic content (51.44 mg GAE/g) and total flavonoid content (2.47 mg RE/g) (22). High antioxidant amino acid residues can be obtained from queen bee larvae (QBL) using simple processing methods (enzymatic hydrolysis and membrane isolation) (107).

Honeybee larvae could also use as potent clinical medicines or functional food for tumor therapy (22) and an adjunctive therapy for the management of atopic dermatitis (AD) (108).

### Summary

- The content of the nutritional components in the three kinds of the bee products are different;
- Carbohydrate are mostly studied in the BCP; Lipids are most studied in the honey, beeswax and BCP; Protein are most studied in the propolis, BCP, RJ, BV; the peptide are most studied in the BV;
- The carbohydrates are the most content in the honey, contributes 95–97% of its dry weight; Fresh RJ contains (60–70% w/w) water. The dry matter of the RJ is composed of protein (27–41%), carbohydrates (~30%), lipids (8–19%); peptides are the main components in BV, ~48–50% of dry venom were the small proteins and peptides; Propolis is composed of 45–55% vegetable balms and resins, 8–35% wax, 5–10% essential oils and aromatics, 5% pollen, 5% fatty acids, and 5% other organic compounds, and minerals; BCP contains about 15–60% carbohydrates, total lipid content of the BCP varying from 1 to 13% from different botanical species, proteins are the second largest amount of nutritional component in BCP and fulfill the nutritional requirements of a honeybee. The content in the larvae are different according to the stages.

All the bee products are potent clinical medicines or functional food for human being.

## Storage of the Bee Products

Owing to the high moisture level in the BCP and BB, the process of dehydration is essential since this moisture causes rapid fermentation and deterioration (109). The vitamin contents of pollen can be altered by processing and storage (61, 110). The quality of the product for consumption, from the monitoring of its production, preparation, and storage should be clarified to ensure the beneficial actions of the bee products.

### Bee Pollen and Bee Bread

For fresh BCP with high moisture levels, storage conditions can affect much of the nutrients it contains. The BCP is dried (111) or pulverized (112), freeze-dried (9, 62), treated with vacuum to remove the impurities, then packed in glass bottles and stored at −18°C (9, 111, 113–115), −20°C (62, 116, 117), 4°C (36, 112, 118) or stored in sterilized containers or bags and stored at 4°C (119, 120), in the dark at room temperature (±20°C) (121). Some researchers also homogenized the BCP samples and stored them under argon at −24°C (122). *P. mirabilis* was found after 1- and 2-year vacuum cold storage (0–4°C) in dried flower BCP (123). It is possible to infer that it is better to consume BCP frozen at −20°C compared with that dried in an electric oven (124).

Studies have shown that pure dried BB, fresh BCP and all conserved mixtures with dried BB, fresh BCP can be stored in a refrigerator at 4–5°C for 2 years and at room temperature for 1 year (34, 125) without any marked changes in the product quality. Other researchers have also chosen to store bee bread at a lower temperature. Usually, samples are put in a freezer and conserved at −12°C (126). After lyophilization and stored at −20°C (30, 127).

### RJ and Honey

Refrigeration and freezing can delay and effectively reduce the chemical changes in RJ during storage, and thus escape the effect of the heat, light, and air. Regarding the shelf-life, Donadieu (128) recommended that the optimal conservation temperature is 0–5°C for fresh RJ preservation for 1 year, whereas the lyophilized RJ can store at room temperature for several years without deterioration (129). A recent study from the MARIA CRISTINA MESSIA who determined the Furosine of the freezed drying RJ during different days and temperature, and pointed out that the lyophilized RJ was more prone to Maillard reaction than fresh RJ for the furosine values risen during the days passing by, even 18 months later. Researchers also pointed that the storage temperature of 4°C is appropriate for raw RJ (130).

The storage temperature was 4°C, the shelf life of the fresh RJ was 6 months, while for lyophilized RJ, or lyophilized RJ in honey (humidity <18%), and the Apilac pills (lactose-glucose-freeze-dried RJ), the time would be 1 year, 2 year at room temperature, and at 4–8°C for 2 years, the shelf lives of fresh RJ and lyophilized RJ were 2 and > 2 years, the temperature was set at < −18°C, respectively (131). BCP, propolis, honey, and BB were stored at 6°C for a maximum of 4 weeks, while for RJ was stored at −18°C (52, 132). The collected honey samples can be stored at 4°C (133, 134), or 20°C (135).

### Propolis

Raw propolis was stored at 4°C (136). To ensure the propolis quality, the samples were harvested using a plastic propolis trap and stored in the dark at −20°C (~30 days) (15), or even grounded in a marble mortar at −30°C (137).

### Summary

- The storage condition is different according to the bee products;
- Fresh BCP and BB with high moisture are easy contaminated by the microbial infection; Dried BCP and BB are better store at lower temperature in the dark at room temperature; the storage condition should be choose store at −18, −20, 4°C or stored in sterilized containers or bags and stored at 4°C in the dark at room temperature (±20°C), homogenized the BCP samples and stored them under argon at −24°C;
- Raw propolis was better stored at lower temperature.
- The collected honey samples can be stored at 4 or 20°C;
- The storage temperature for the RJ is controversy for the water content in it; storage temperature of 4°C is appropriate for raw RJ, for the lyophilized RJ temperature could be at room temperature, and at 4–8 or < −18°C for different days.

## Processing Techniques Applied in Bee Products

**Figure 3:**
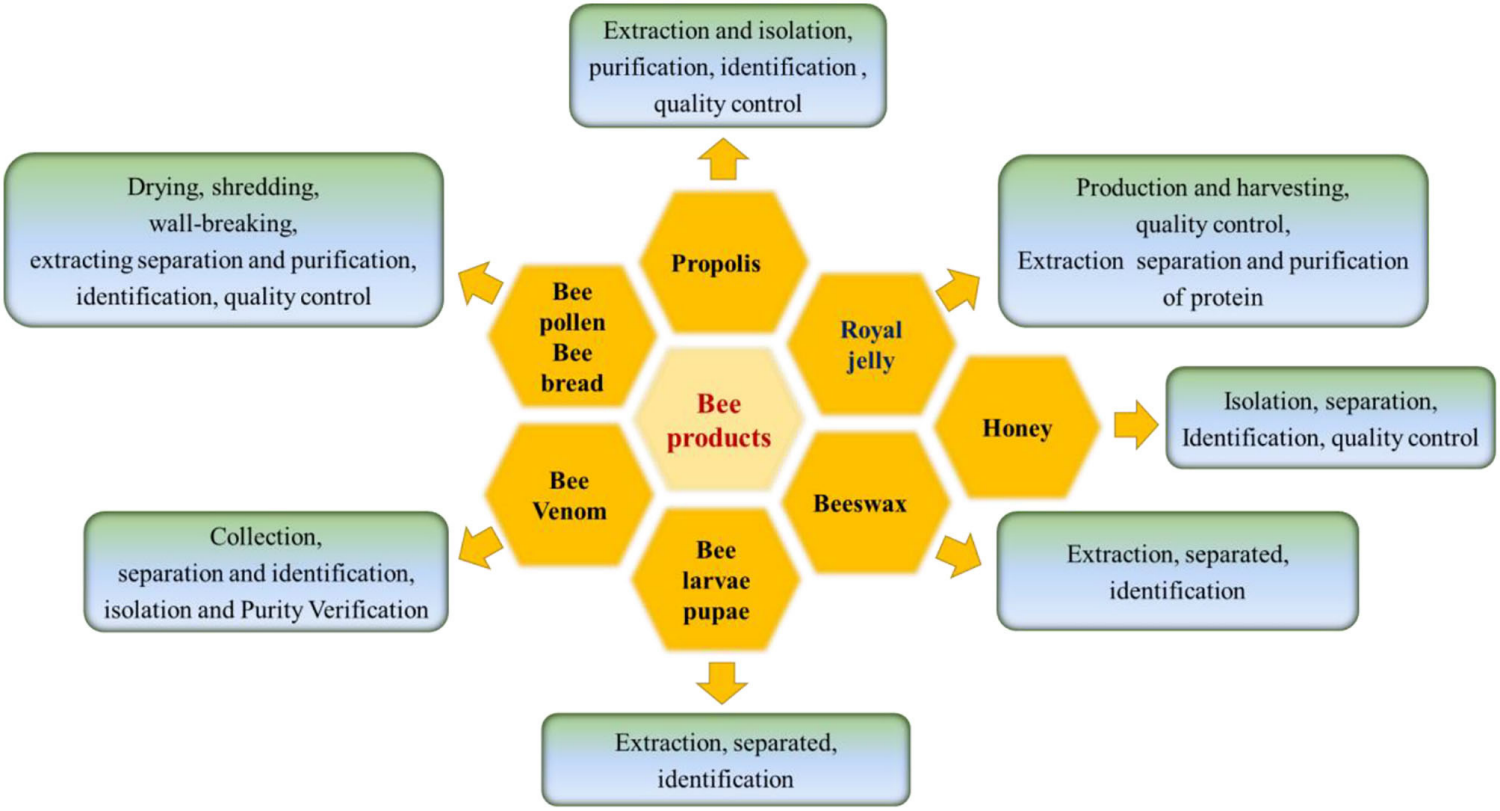
Processing techniques used for bee products.

**Figure 4:**
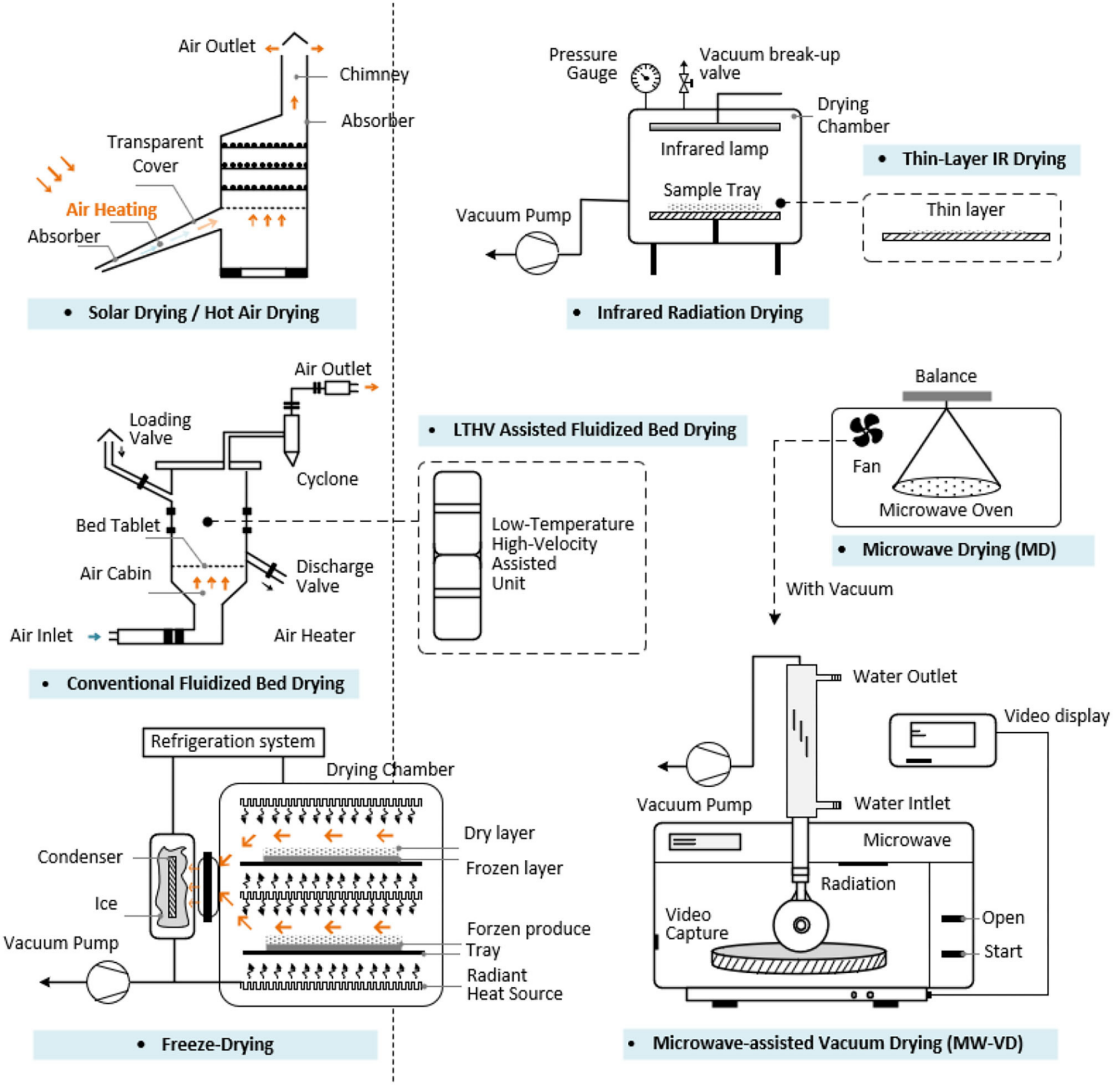
The effects of each drying techniques.

Processing techniques influence food composition and structure, including the flavor, physical properties, functions, and in turn the product quality (138). The processing techniques applied in the bee products are shown in the Figure 3. Drying techniques is essential for controlling the moisture of BCP and BB after harvest. For RJ, the production and harvest are distinct from that of other bee products as the machines used are different. Special techniques with minimum impact on the health of bees are needed to collect a considerable quantity of BV (139). For the other bee products, extraction, separation, purification, and identification procedures are in common. Quality control techniques are important applied in BCP, BB, RJ, honey, and propolis. The characters and the effects of each drying techniques are shown in Supplementary Table 2 and Figure 4.

### Drying Techniques

Fresh BCP contains ~20–30% water. The higher humidity makes BCP easily contaminated by microorganisms such as bacteria and yeast. To prevent spoilage and preserve the maximum quality, the BCP must be harvested daily and frozen immediately. The drying of BB is also an important technological process that affect the quality of the bee product (140). Among the four steps procedure (drying, segmenting, filtering, and disinfecting) (33) for the BB industrial collection, drying is the most important process.

#### Traditional Drying Techniques

Traditional drying methods include freezing, solar drying and in a hot air chamber. The bioactive compound content like carotenoid and phenol content in dry BCP and the antioxidant activity (0.85 mmol Trolox/g) did not change substantially from the initial raw BCP drying by solar dehydration (141).

Compared with the sun drying, hot air drying has a reasonably shorter processing time, more effective sanitary conditions, lower risk of microbial contamination, and better control of drying conditions. It has been used for the dehydration of food and agricultural products in an industrial scale drying applications (118). But hot air drying processing has an effect on the BCP quality and organoleptic characteristics, physicochemical properties, morphological structure (118). BCP dried at 40 and 50°C showed a marked increase in acidity, flavonoids, phenolics, and antioxidant activity after thermal treatment, and a loss of carotenoids, while the most adequate temperature for BCP drying is at 60°C (142).

In comparison with the convective drying, the cyclic convective drying of Perga is not only highly effective to reduce the moisture content and can also reduce energy costs by more than two times. The low speed of the cyclic drying process avoids overheating of the Perga and ensures the safety of biologically active substances (143).

FD is considered the most gentle drying method, with fast rehydration rates and high rehydration capacity, as it causes negligible damage to the product microstructure and thus maximum preserve the physical and chemical properties (144). FD influenced the rutin level of the honeybee-collected chestnut pollen, when treated for 420 and 540 min, rutin and amino acid parameters were maintained in the range that required for the standardization of commercial BCP in the European market (145).

Currently, the freezing and FD methods are often used to preserve RJ. The optimal mathematical model was build and suitably used for calculating and setting up the technological mode of the RJ FD processing (146). During the FD process, the nutritional properties of RJ well-preserved, but FD RJ is highly susceptible to Maillard reaction (MR) and cannot meet the product quality and stability requirements (130). The final product of the FD process of RJ had a very good quality (147).

#### Innovative Drying Techniques

IR radiation drying is an innovative drying technique which can be applied in BCP samples. IR radiation greatly affected the drying and quality characteristics of the samples, especial the color. Surface morphology changes of BCP grains a lot with the increasing IR intensity.

Drying the BCP using IR radiation at 50 W better retained its quality characteristics than treated at other power levels (148). An IR heating-assisted fluidized bed dryer was applied in the BCP drying process to ensure physical and microbiological stability. The drying condition is more smoother than thin-layer IR drying that prevented color degradation, thus conserving product quality of the BCP, and compared with the conventional fluidized bed drying the specific energy consumption was 52% lower (51).

The developed model of BB drying with IR heating or the combine use of the IR heating with the convection drying was built using the COMSOL Multiphysics. The geometric dimensions of the drying chamber, the trays with the product, the thermal and physical properties of air and BB should be considered (140).

Microwave heating can be an efficient drying method. Compared to the conventional drying, microwave can lead to a faster process and obtain better quality of the dried material at the same cost (149). Irradiating fresh BCP with microwaves under vacuum can effectively reduce the water content without thermally deteriorating important bioactive compounds (26). Microwave drying was important for the BCP conservation, but the microwave treatment showed a damaging action on tocopherols (150) and proline content at the highest level treatment (151).

A domestic digital microwave oven (505 mm × 574 mm × 376 mm in size) (Arcelik MD 599, Turkey) with technical features of 230 V and 50 Hz was used for the BCP drying. Operating at a frequency of 2,450 MHz with 180 W microwave power level retained its quality (152). BCP dried using microwave-assisted vacuum drying (MW-VD) had higher antioxidant activity than hot air drying method (HAD), irrespective of the pressure or power level (153).

Different drying processes (FD, MWD, and HAD) affect the polyphenol, flavonoid, and amino acid contents of the BCP (chestnut, willow, ivy). All drying techniques led to a depletion of flavonoids in willow BCP; Whereas, FD and MWD did not affect the flavonoid content in the ivy pollen during storage, the amino acid-related quality of BCP was efficiently preserved up to 6 months. MWD ensured the highest flavonoid content even after 6 months (154). However, low temperature high velocity (LTHV)-assisted fluidized bed drying method preserve the bioactive components of BCP (155), it can be applied especially drying BCP as a bioactive food at 4°C.

### Wall-Breaking

**Figure 5:**
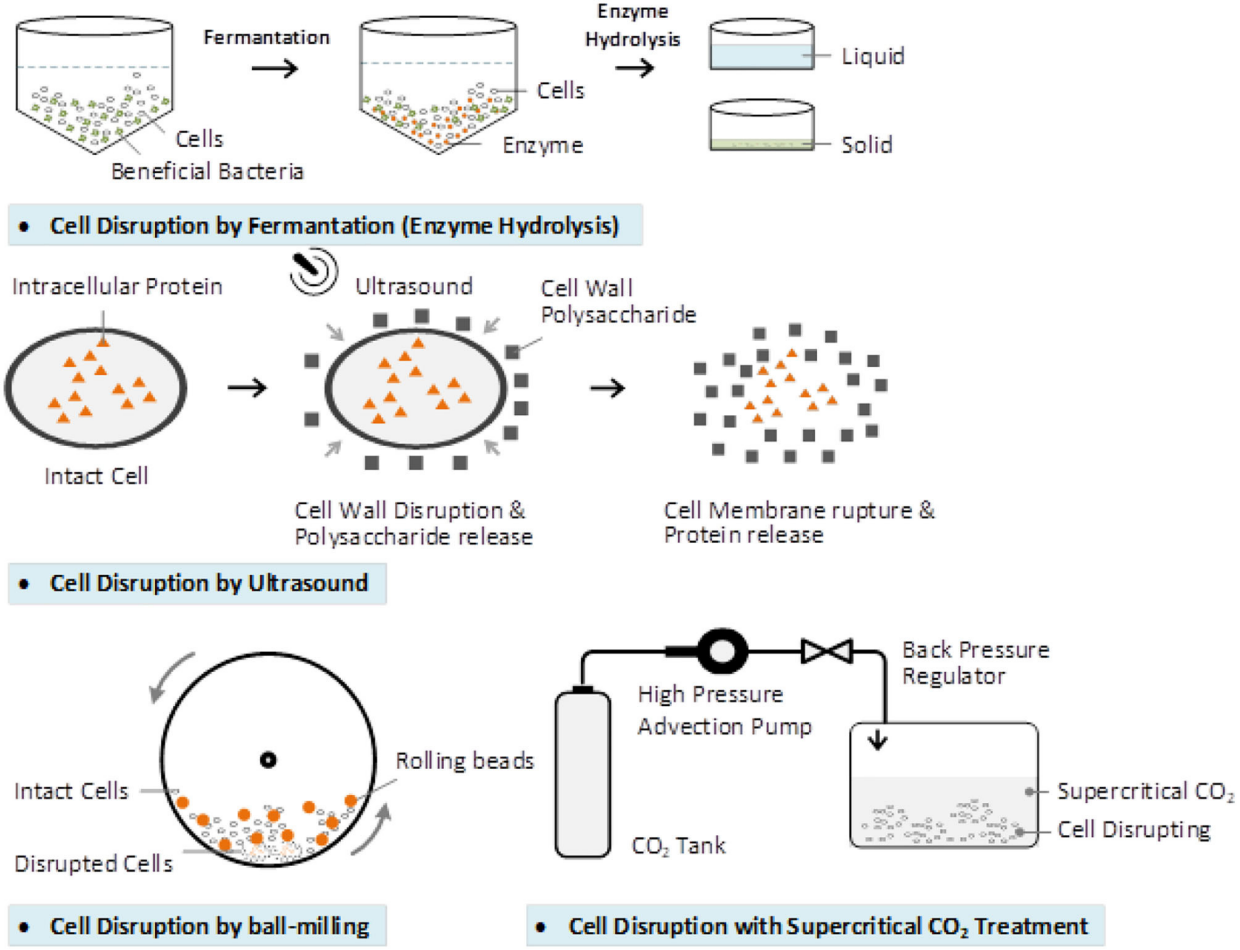
The mechanism of wall-breaking techniques.

BCP cell wall contains two distinct layers. The outer layer exine is made of sporopollenin, which provides chemical resistance and preserves the bioactive compounds in it. The inner layer is intine, which is composed of cellulose and similar to that of a plant cell wall (156). Wall breaking methods like physical, chemistry, biological and combinations are used to break the BCP cell wall (157). The effects of each of the BCP cell wall breaking and some related research are shown in the Figure 5 and Table 1.

**Table 1:** BCP wall-breaking.

**Bee products**	**Wall-breaking method**	**Conditions and results**	**Advantages and disadvantages**	**References**
Rose BCP (China)	Ball-milling treatment	♢ Milling for 3 min at 25 Hz frequency		(29)
Rose BCP (China)	Ultrasonic treatment	♢ At acoustic power of 600 W and frequency of 20 kHz treating 4 h at 25°C	➢ More effective in nutritional components release ➢ Causes more losses in vitamin B1 and vitamin C	(29)
Rape BCP (China)	Supercritical carbon dioxide	♢ More effective with higher CO_2_ pressure ♢ Optimum oil yield was obtained 5.98% of dry BCP ♢ Optimum conditions: 39.2 MPa and 54.7°C and CO_2_ flow rate of 17.1 L/h after treated with supercritical CO_2_ for 2 h.	➢ Under low temperature with short time ➢ Higher selectivity ➢ No solvent residue in the extract	(156)
Rape BCP, *Brassica campestris* L., BP (China)	Microbial fermentation (lactic acid bacteria, yeast, the two mixed)	♢ 83.5% reduction in fructose and 87.4% glucose reduction after yeast-fermented ♢ Oligopeptides, fatty acids and phenolic compounds increased by 68.8, 18.2, and 9.3%		(158)
Bee-collected pollen (BCP) flowers of ivy (Italy)	Fermented with selected microbial	♢ Incubated kunkeei strains and H. uvarum AN8Y27B at 30°C for 216 h	➢ Markedly increased the number of volatile compounds (VOC)	(159)
BCP (Colombia)	Enzymatic hydrolysis	♢ Proteases improved the protein content by ~13–18%, phenolics 83–106%, flavonoids 85–96%, ♢ increased all essential amino acids and antioxidant activity up to 68%		(160)
Pollen *Cistus ladanifer* (Japan)	Enzymatic hydrolysates	♢ Three types of enzymes (pepsin, trypsin, and papain) ♢ high content of proteins and phenolic compounds after enzymatic hydrolysates		(161)

*BCP, Bee Collected Pollen; VOC, volatile compounds; CO_2_, carbon dioxide*.

Several biotechnological solutions have been proposed for BCP processing to increase the accessibility to nutrients for intestinal absorption, the fermentation being one of the most potential method. Fermented BCP is enriched in bio accessible phenolics with recognized bioactivity in humans (159). The yeast-fermented BCP greatly increased the number of active ingredients such as phenolic compounds, nicotinic acid, oligopeptides, nicotinamide, fatty acids, riboflavin, and free amino acids (158). Fermented BCP is distinguished by its higher nutritional value and better digestibility because the BCP cell walls are partly destroyed during the fermentation process (69).

In addition to the role of microorganisms, the use of proper enzymes is a good way to hydrolyze the wall of the BCP. Enzyme hydrolysis is safe, inexpensive, and easy to apply. Pepsin, trypsin, and papain hydrolysates from BCP are beneficial not only in health food diets, but for special patients with diseases as cancer, cardiovascular, and diabetes. Thus, honeybee-collected pollen peptides can be used in various fields of medicine and food technology (161). Compared to other BCP modification strategies, proteases improved the BCP protein content by ~13–18%, phenolics 83–106%, flavonoids 85–96%, and all essential amino acids were increased, even the antioxidant activity increased up to 68% (160).

Physical processing technology also resulted in the breaking of the BCP cell wall. Ultrasonication and high shear (US-HS) technique was applied to break the cell walls of five species of BCP. The digestibility of protein and crude fat, amino acids, and reducing sugars increased to more than 80% and nearly 1.5-and 2 times, respectively (162). Besides, an ultrasonic temperature difference was applied to treat the bee pollen under specified conditions: the ratio of water to material 20 mL/g, ultrasonic power 400 W, temperature difference 90°C, ultrasonic treatment time 40 min. The extracts after the wall-breaking are potential to be developed as effective functional ingredients as immunomodulatory (163).

Ultrasonic and ball-milling treatment especially the ultrasonic treatment affected nutritional component release and thus change the antioxidant activities *in vitro* and *in vivo* of the rose BCP (29). Supercritical carbon dioxide (CO_2_) could be a feasible and a promising cell wall-breaking method applied to the BCP (156).

### Other Processing Techniques

#### Shredding of the Bee Pollen

The technology of shredding was optimized in the BCP to obtain high-quality powder by its technological and physical-chemical indicators. By using modern types of shredders, it is possible to shred BCP to particles with sizes ranging from 120 to 8 μm. Technological parameters of shredding BCP in a mill-mortar: speed is 70–80 rpm (min^−1^), treatment duration is 6 min, and the batch weight is 150 g (164).

#### Collection and Membrane Ultrafiltration of BV

To collect a considerable quantity of BV, Markovic and Molnar (1963) used electroshocks and squeezed to induce a bee sting (165). A method modified from Benton et al. (166) was used to collect the liquid fraction of the BV in sufficient quantity to facilitate chemical analysis. Carpena et al. also used electroshock traps to collect more BV (139). BV from free-living honeybees (*A. mellifera*) was obtained using a collecting device (Chung-Jin Biotech Ltd., Ansan, Korea) or Bee Whisper 0412 BV collector (IGK Electronics, Bulgaria). The collectors were installed at the entrance of the hive to collect crude BV (167, 168).

Hermia's models were used to predict the fouling mechanisms during cross-flow ultrafiltration (UF) (169) of BV in a 10 kDa regenerated cellulose membrane (170, 171).

#### Production and Harvesting of RJ

The conventional method of producing RJ usually involves five steps: cleaning queen cells, grafting larvae, inserting RJ production frames, supplementary grafting, and harvesting. A new method based on a device that obviates grafting and is therefore convenient and efficient (172, 173).

Currently, electroshock technology is widely used for the BV collection. For the RJ production technology, the new method is convenient and efficient.

### Extraction, Separation, and Purification Techniques

Considering the current demand for healthy and natural food, some hive products have been attracting the commercial interest recently, for several health-promoting compounds contained, making them one of the most widely consumed food supplements. Extraction and separation techniques are essential for obtaining large quantities of high-quality active ingredients. Recent study (5 years) about the sugars/polysaccharide extraction, separation and purification techniques were shown in the Table 2.

**Table 2:** Sugars/polysaccharides from bee products (2017–2021).

**Bee products** **reference**	**Sugars or polysaccharides**	**Extraction method**	**Determine method**	**Separation/purification**	**Identification/analysis**
Honey (France) (174)	–			SPE filtered on 0.45 μm syringe filter	HPAEC-PAD Analysis
BCP (China) (20)	–		Phenol-sulfuric acid method	DEAE-52 cellulose column	Fourier transformed-infrared (FT-IR)
BCP (China) (175)	–	90% ethanol solution; Next soaked in distilled water	Phenol-sulfuric acid		Gas chromatography
BCP (China) (163)	–	Temperature-difference ultrasonic treatment; extraction was carried out at 85°C		Quadruple volume of absolute ethanol precipitates	
BCP (China) (176)	Arabinose, xylose, galactose, mannose, ribose, rhamnose, galacturonic acid, glucose, and in a molar ratio of 1.03: 0.08: 0.67: 0.38: 0.09: 0.17: 0.64: 0.22	With distilled water extracted three times at 85°C for 2 h each	Phenol-sulfuric acid	A quadruple volume of absolute ethanol precipitates	High-performance gel permeation chromatography
BCP of *Nelumbo nucifera* (China) (177)	Rha (11.5%), Ara (29.7%), GalA (12.0%), Gal (41.2%), 1.5-L-Ara (25.6%), 1,6-D-Gal (18.3%), t-D-Gal (12.0%)	Hot water extraction		DEAE-cellulose, Sepharose CL-6B	GC-MS 13C-NMR
BCP (China) (178)	Two sub-fractions: RBPP-N and PBPP-P (yield 23.4% and 35.8%) and PBPP-P (yield 35.8%); Glc (glucose 35.0%) is special contained in RBPP-N, which might be glucan and arabinogalactan (AG); Ara (arabinose: 37.8%, 51.8%), and Gal (galactose: 27.2% and 21.0%) are identify out in both subfractions, Rha (rhamnose: 5.4%), and Gal A (galacturonic acid: 18.9%) were special contained in RBPP-P, might be monosaccharides for pectin;	Hot water extraction and ethanol precipitation		DEAE-cellulose	13C-NMR, FT-IR spectroscopy
RJ (Greece) (132)	Glucose, fructose, and sucrose			Zorbax Carbohydrate Analysis Column	HPLC-RID technique
Honey (European) (China) (179)	**Monosaccharides** (glucose, fructose, rhamnose, mannose); **Disaccharides** (sucrose, -trehalose, turanose, maltose, isomaltose, gentiobiose nigerose, maltulose, palatinose, melibiose, melezitose, and kojibiose); **Trisaccharides** (melezitose, maltotriose, panose, raffinose, isomaltotriose, erlose, and 1-kestose).				NMR
Honey (Italy) (133)	Seven monosaccharides; Four trisaccharides; eight Disaccharides; One tetrasaccharide;			CarboPac PA10™ column (Thermo Scientific, 2 × 250 mm, 10 μm) equipped with a CarboPac PA10™ guard column (2 × 50 mm)	High-performance anion-exchange chromatography coupled to a mass spectrometry detector
BCP (Slovenia) (9)	Glucose; Fructose; Maltose; Sucrose; Melezitose			Solid-phase extraction (SPE); HPLC column (100 × 2.1 mm internal diameter; 3.5 μm particle size; Waters XBridge Amide)	Triple quadrupole mass spectrometer
BB (Morocco.) (30)	Fructose; Glucose; Trehalose	96% ethanol ultrasonic bath;			HPLC coupled to a RI detector

*BCP, Bee Collected Pollen; BB, Bee Bread; RJ, Royal Jelly; HPAEC-PAD, High Performance Anion Exchange Chromatography-Pulsed Amperometric Detector; FT-IR, Fourier transformed-infrared; GC-MS, Gas Chromatography-Mass Spectrometer; 13C-NMR, 13 C nuclear magnetic resonance; HPLC-RID, High Performance Liquid Chromatography Refractive Index Detection; DEAE, Diethylaminoethyl cellulose*.

#### Sugars/Polysaccharide

Carbohydrate was the largest fraction that contained in the honey and BCP. The concentration and carbohydrates ratio may play a role in the identification of geographical origins and the monofloral of the honeys (180). The main compound in honey is carbohydrates, which contribute about 95–97% of its dry weight (41, 48). Predominantly, monosaccharides such as glucose and fructose (40, 49, 50, 181), mannose, and rhamnose are common in honey. In addition, disaccharides, trisaccharide, and tetra saccharides have also been identified in honey (133, 179). The beneficial effects of honey on human health have long been recognized (180). BCP contains about 15–60% carbohydrates, which represent the most fraction in the BCP (182). Monosaccharides are the major source of metabolism energy, polysaccharides store energy, and are also the structural components (183). Glucose and fructose are the most abundant reducing sugars in BCP (9). In addition, other carbohydrates such as disaccharides (sucrose), monosaccharides (pectin, glucan, and arabinogalactan), oligosaccharides, and dietary fiber were also found in BCP (176, 178). The main sugars in RJ are fructose and glucose in relatively constant proportions similar to those of honey (13).

The extraction methods of polysaccharides focus on the traditional method, after 96% ethanol (175) or ultrasonic assist (30) solvent to skim the lipid, hot water (163, 176–178) is used to extract the polysaccharide, and then a quadruple volume of absolute ethanol precipitates (163, 176, 178). Solid-phase extraction (9, 174) as well as DEAE-52 cellulose column (20, 177, 178), Zorbax Carbohydrate Analysis Column (132) and other columns were used to separate the polysaccharides. High-performance liquid chromatography (HPLC), HPAEC-PAD (174), Fourier transform infrared (FT-IR) spectroscopy (20, 178), gas chromatography (GC) (175), high-performance gel permeation chromatography (176), GC-MS (177), ^13^C NMR (177–179), and HPLC-RID (132) techniques were used to identify the polysaccharides.

#### Lipid or Fatty Acids

Besides the carbohydrates and proteins in the BCP, lipids are the third-largest constituent, lipids are vital for the generation of RJ (3). Among bee products, lipids from BCP, BB, RJ, and Beeswax have been widely studied in recent 5 years. The fatty acids identified are listed in Table 3. Lipids from bee products extracted using a Soxhlet extractor (62, 186) or extracted with a simple solvent. The extraction solvents such as petroleum ether (124), hexane:isopropanol (184), and diethyl ether:petroleum ether (2:1, v/v) (186, 187) diethyl ether/iso-propanol, chloroform:methanol (1:1), chloroform:isopropanol (4:1) (150), methanol/diethyl ether/isopropanol 50/1 (v/v) (188), heptane (112), (CH_2_Cl_2_:MeOH) (2/1 v/v) extraction (189), and n-hexane (111, 127, 190) are used these years. Gravimetric method was used to determine the total lipid content in all cases.

**Table 3:** Lipid extraction, separation purification, and identification of the fatty acids from the bee products (2017–2021).

**Bee products** **reference**	**Compounds**	**Extraction method**	**Separation/purification**	**Identification/analysis**
BCP (Italy) (184)	Ten saturated and the other unsaturated C8:0; C13:0; C15:0; C16:0; C17:0; C18:0; C18:1; C18:2n-6t: C18: 2n-6c; C18: 3n-6; C18: 3n-3; C20:0; C20:1; C20:2; C22:0; C22:1; C23:0; C24:0 N-6PUFA; n-3 PUFA	Hexane:isopropanol	Silica Gel 60, 230/400 mesh	Thin-layer chromatography Gas chromatography
BCP (China) (185)	Phospholipids and polyunsaturated fatty acids	Folch method	XSelect CSH C18 100 × 2.1 mm, 2.5 μm column (Waters) and CORTECS C18 100 × 2.1 mm, 2.7 μm column (Waters)	UPLC-Q-Exactive Orbitrap/ MS
BCP (Portugal) (124)	–	Petroleum ether using an automatic Soxtec device	Gas chromatography	Flame ionization
BCP (Italy), (Spain), (Colombia) (186)	–	Soxhlet extraction using a Soxtec HT 1043 system ether:petroleum ether (2:1, v/v)	Omegawax 320 capillary column;	GC-FID
RJ (Italy) (187)	–	Diethyl ether/iso-propanol	Fused silica capillary column SE-52	Capillary Gas Chromatography GC/MS
BCP (Brazil) (57)		Soxhlet extraction		
BCP (Italy) (150)	(150) ω-6 fatty acidsω-3 fatty acids and carotenoids	Chloroform:methanol (1:1) solution solid-phase extraction Chloroform: isopropanol (4:1)		GC2010 Shimadzu gas chromatograph High polar fused-silica capillary column
RJ (Greece) (188)	10-DHA; 2-Dodecenedioic acid; Decanoic acid; Decanedioic acid; 3-Hydroxydecanoic acid; Decanedioic acid;	Methanol/ Diethyl ether/isopropanol 50/1 (v/v)		Liquid chromatography-high resolution MS
BCP/BB (Turkey) (113)	Caproic acid; Caprylic acid; Myristic acid; Palmitoleic acid; Stearic acid; Oleic acid; Linoleic acid; Linolenic (ALA) acid; Arachidic acid; Eicosenoic acid; Eicosadienoic acid; Behenic acid; Erucic acid; Tricosanoic acid; Lignoceric acid; Palmitic acid; Nervonic acid; Docosahexaenoic acid; Capric acid; Lauric acid; Heneicosanoic acid; Heptadecanoic;	Ethanol (95%), ultrasonic-assisted extraction; AOAC method Soxhlet extraction	A DB-23 60 m × 0.25 mm ID, 0.15 μm (J&W 122–2,361) column	GC and 5,973 MSD mass spectrometry
BCP (Serbia) (112)	C18:0; C12:0; C14:0; C15:0; C16:0; C16:1; C17:0; C18:0; C18:1n-9; C18: 2n-6; C18: 3n-6; C20:0; C21:1; C20:2; C20:3n-6; C20:3n-3; C22:0; C22:5n-3; C24:0;	Ultrasonic assist heptane extraction for 15 min	Filtered through filter paper (Whatman No. 2); cyanopropyl HP-88 column	GC capillary analysis with FID
BCP (Korea) (62)	C6:0; C8:0; C10:0; C12:0; C13:0; C14:0; C15:0; C16:0; C16:1; C17:0; C18:0; C18:1; C18:2 n-6; C18:3 n-6; C18:3 n-3; C20:0; C20:1; C20:2; C20:3 n-3; C20-4 n-6; C21:0; C22:0; C22:1; C22:2; C22:6 n-3; C23:0; C24:0; C24:1;	Soxhlet extraction		GC-FID
BCP (Turkey) (114)	Lauric; Myristic; Palmitic; Linolelaidic; Arachidic; Linolenic; Behenic; Arachidonic Stearic; Elaidic; Oleic; Linoleic;		FID and capillary column	GC
BCP (Malaysia) (36)	Butanedioic acid, Butanoic acid, bis(trimethyl silyl) ester; 4-[bis(trimethylsilyl) amino]-, trimethylsilyl ester; Linoleic acid ethyl ester; ethyl ester;9,12-Octadecadienoic acid (Z, Z)-, trimethyl silyl ester; alpha.-linolenic acid, trimethyl silyl ester; Dodecanoic acid, tetradecyl ester; 9,12,15-Octadecatrienoic acid;	Ethanol extraction solicited in an ultrasound bath	Agilent mass selective detector (MSD) 5975C, HP-5 MS fused-silica capillary column (30 m × 0.25 mm I.D, 0.25 μm film thickness)	(GC-MS)
BCP (India) (111)	C4:0 Butyric acid; C6:0 Caproic acid; C8:0 Caprylic acid; C10:0 Capric acid; C11:0 C12:0 Lauric acid; C14:0 Myristic acid; C15:0 Pentadecanoic acid; C16:0 Palmitic acid; C16:1, cis-Δ7 (ω-7) Palmitoleic acid; C17:0 Heptadecanoic acid; C17:1, cis-Δ7 (ω-7) Heptadecenoic acid; C18:0 Stearic acid; C18:1, trans-Δ9 (ω-9) Elaidic acid; C18:1, cis-Δ9 (ω-9) Oleic acid; C18:1, cis-Δ6 (ω-12) Petroselinic acid; C18:2, cis, cis-Δ9,12 (ω-6) Linoleic acid; C18:2, trans, trans-Δ9,12 (ω-6) Linoelaidic acid; C18:3, cis, cis, cis-Δ9,12,15 (ω-3) α-Linolenic acid; C18:3, cis, cis, cis-Δ6,9,12 (ω-6) γ-Linolenic acid; C20:0 Arachidic acid; C20:1, cis-Δ11 (ω-9) Eicosenoic acid; C21:1, cis-Δ9 (ω-9) Heneicosanoic acid; C20:2, cis, cis-Δ11, 14 (ω-6) Eicosadienoic acid; C20:3, cis, cis, cis-Δ8, 11, 14 (ω-6) Dihomo-γ-linolenic acid; C20:3, cis, cis, cis-Δ11,14,17 (ω-3) Eicosatrienoic acid; C20:4, cis, cis, cis, cis-Δ5,8,11,14 (ω-6) Arachidonic acid; C22:0 Behenic acid; C22:1, cis-Δ13 (ω-9) Erucic acid; C22:6, cis, cis, cis, cis, cis, cis-Δ4, 7, 10, 13, 16, 19 (ω-3) Docosahexaenoic acid; Undecanoic acid; C23:0 Tricosanoic acid; C20:5, cis, cis, cis, cis, cis-Δ5,8, 11, 14,17 (ω-3) Eicosapentaenoic acid; C24:0 Lignoceric acid; C24:1, cis-Δ15 (ω-9); Nervonic acid;	n-hexane extraction in a Soxhlet apparatus for 8 h	SLB-IL111 capillary column (100 m × 0.25 mm internal diameter, 0.20 μm film thickness; Sigma-Aldrich)	GC-FID
BB (Romania) (127)	C4:0 Butanoic acid; C6:0 Hexanoic acid; C8:0 Octanoic acid; C10:0 Decanoic acid; C11:0 Undecanoic acid; C12:0 Dodecanoic acid; C13:0 Tridecanoic acid; C14:0 Tetradecanoic acid; C14:1 (cis-9) (Z)-tetradec-9-enoic acid; C15:0 Pentadecanoic acid; C15:1 (cis-10) (Z)-pentadec-10-enoic; C16:0 Hexadecanoic acid; C16:1 (9Z)-hexadec-9-enoic acid; C17:0 Heptadecanoic acid; C17:1 cis-10-heptadecenoic; C18:0 Octadecanoic acid; C18:1 (E)-octadec-9-enoic; C18:1 (Z)-octadec-9-enoic; C18:2 (all-trans-9,12) Octadeca-9,12-dienoic acid; C18:2 (all-cis-9,12) (9Z,12Z)-octadeca-9,12-dienoic acid; C18:3 (all-cis-9,12,15) Octadeca-6,9,15-trienoic acid C20:0 Icosanoic acid; C18:3 (all-cis-6,9,12) Octadeca-6,9,12-trienoic acid;C20:1 (cis-11) (Z)-icos-11-enoic acid;; C20:2 (all-cis-11,14,) Icosa-11,14-dienoic acid; C20:3 (all-cis-8,11,14) (8Z,11Z,14Z)-icosa-8,11,14-trienoic acid; C20:3 (all-cis-11,14,17); (11Z,14Z,17Z)-icosa-11,14,17-trienoic acid; C20:5 (all-cis-5,8,11,14,17); (5Z,8Z,11Z,14Z,17Z)-icosa-5,8,11,14,17-pentaenoic; C20:4 (all-cis-5,8,11,14); (5Z,8Z,11Z,14Z)-icosa-5,8,11,14-tetraenoic acid; C21:0 Heneicosanoic acid; C22:0 Docosanoic acid; C22:1 (cis-13) (Z)-docos-13-enoic; C22:6 (all-cis-4,7,10,13,16,19); Docosa-4,7,10,13,16,19-hexaenoic; C22:2 (all-cis-13,16) Docosa-13,16-dienoic; C23:0 Tricosanoic acid; C24:0 Tetracosanoic acid; C24:1 (cis-15) (Z)-tetracos-15-enoic;	Extracted with n-hexane		GC-MS
BB (Morocco.) (30)	C8:0; Octanoic; C10:0 Decanoic; C11:0 Undecanoic; C12:0 Dodecanoic; C13:0 Tridecanoic; C14:0 Tetradecanoic; C15:0 Pentadecanoic; C16:0 Hexadecanoic; C16:1 Hexadecanoic; C17:0 Heptadecanoic; C18:1n9 Oleic; C18:2n6 Linoleic; C18:3n3 α-linolenic; C18:0 Stearic; C20:0 Arachidic; C20:1 Gadoleic; C20:2 Eicosadienoic; C20:33n6 Eicosatrienoic; C20:4n6 Arachidonic; C20: 3n3 Eicosatrienoic; C22:0 Behenic; C20:5n3 Eicosapentaenoic; C24:0 Lignocerin; C24:1 Nervonicacid;			GC-FID
Beeswax (189)	C12:0, C13:0, C15:0, C16:0, C18:0, C20:0, C24:0, C25, C16:1, C20, C27, C28, C29, C30, C31, C32, C33, C36; C14:0, C26, C18:1, C23;	Ultrasonic added dichloromethane/methanol (CH_2_Cl_2_: MeOH) (2/1 v/v) extraction		GC; GC/FID; GC–MS
BCP (Algeria) (190)	Lauric acid; Sebacic acid; Palmitic acid; Unidentified; Linolenic acid; Linoleic acid; Oleic acid; Stearic acid; Myristic acid; Tricosanoic acid; 17-Pentatriacontene; Arachidic acid;	Hexane extracted		GC/MS

*BCP, Bee Collected Pollen; BB, Bee Bread; RJ, Royal Jelly; GC-FID, Flame ionization Detector; GC-MS, Gas Chromatography-Mass Spectrometer; AOAC, Association of Official Analytical Chemists*.

Traditional extraction techniques with the disadvantages as the long extraction time, loss of the volatile compounds, toxic solvent residues, and the unsaturated compounds degraded due to heat (191). In certain cases, it can be resolved by using supercritical fluid (carbon dioxide) extraction (SFE).

For the identification technologies, thin-layer (184), capillary gas chromatography, gas chromatography (111, 114, 190), GC-FID (16, 30, 186, 190), GC/MS (36, 127, 187, 190, 192), GC, and 5,973 MSD mass spectrometry (113), are the preferred techniques for determining fatty acids. The samples detected using GC-MS should be firstly converted into their corresponding methyl esters (FAMEs) using a transesterification procedure, chromatographic data obtained are more robust and reproducible (193).

Total lipid content of the BCP varying from 1 to 13% from different botanical species. The lipid fraction of BCP contains mainly carotenoids, sterols, and fatty acids (194). Fatty acids are important nutritional substances for living organisms, especially those of the ω-3 series. The fatty acid of the BCP with the functional role as an anti-atherogenic food for human metabolism (150). Nearly 20 fatty acids have been reported in BCP from C4 to C24, among which ω-3 fatty acids are dominant (36, 62, 111–114, 150, 184). In beeswax, fatty acids were identified from C12:0 to C36 (189). Approximately 80–90% of the RJ fatty fraction is composed of free fatty acids. The major fatty acid from the RJ were trans-10-hydroxy-2-decenoic acid [10-hydroxydecanoic acid (HDA)], and other four suspect fatty acids decanedioic acid, 2-dodecenedioic acid, decanoic acid, and decanedioic acid were also detected using the liquid chromatography-high resolution mass spectrometry (HRMS) method (188).

#### Proteins, Peptides, and Amino Acids

Bee products such as BCP, BB, and RJ are good sources of protein, which can be consumed as a food supplement to the diet. According to the characteristics of the protein, the extraction solvent usually focuses on distilled water (195), acid extraction (196, 197), phosphate buffer with different pH (pH 7.6) (198), (pH 7.2) (199), or 50 mM Tris-HCl, pH 8 (200). The method for the protein extraction and determination are shown in Table 4.

**Table 4:** The techniques for the protein extraction and determination.

**Bee products**	**Extraction method**	**Determine method**	**References**
RJ		Bio-Rad Protein Assay kit	(19, 201)
BCP (eastern Saudi Arabia)		Micro-Kjeldahl method	(202)
Black Dwarf Honey and BCP (Thailand)		Kjeldahl's method	(203)
RJ (Japan)	Dialyzed against distilled water	Micro BCA Protein Assay Kit (Pierce)	(204)
RJ (Japan)	Dissolved in deionized water and ultracentrifuged	Micro BCA Protein Assay Kit	(195)
RJ (Pisa)	50 mM Tris-HCl, pH 8	Bradford	(200)
RJ (Japan)	Acid extraction		(196)
BCP (Al-Ahsa Eastern Saudi Arabia) (Australia)		Micro-Kjeldahl method using the 5.60 factor	(205, 206)
BCP (China)	Extracted in phosphate buffer pH 7.6		(198)
BCP (*Apis mellifera*) (Thailand)	Pretreated with 1,000 mL of 20 mM phosphate buffer solution (pH 7.2) Hydrolyzed by Alcalase, Flavourzyme, or Neutrase (separately)	Bradford	(199)
BCP (Bulgaria)	Extracted with distilled water the homogenized adjusted pH 7.0 hydrolysis	Kjeldahl using the equation: N×6.25	(207)
BCP		Bradford	(208)
BCP (China)	0.1 mol/L HCl solution		(197)

*RJ, Royal Jelly; BCP, Bee Collected Pollen; BB, Bee Bread*.

In most cases, total protein content was the result of nitrogen measurement using the Bradford (199, 200, 208), Kjeldahl, or Micro-Kjeldahl methods (202, 203), using 5.60 (205, 206) and 6.25 factor (207). In addition, the Micro BCA Protein Assay Kit (195, 204) and Bio-Rad Protein Assay kit (19, 201) were used for protein determination.

The crude proteins were analyzed by sodium dodecyl sulfate polyacrylamide gel electrophoresis (SDS/PAGE) (80, 192, 196, 209–213), and western blotting (19, 201), native-PAGE (209), blue native-PAGE or 2-D blue native/SDS-PAGE (204). The tryptic peptides were subsequently determined using two-dimensional polyacrylamide gel electrophoresis (2D-PAGE), LC-Chip/ESI-QTOF-MS (80, 199), MALDI-TOF/MS (214, 215).

In Tables 4–6, the protein and peptide identified in recent years have been listed. RJ can be consumed by humans as a food integrator, and it has high commercial value because of its nutritional and nutraceutical properties. It is estimated that 50% of dry RJ constitutes of proteins (237). Majority of RJ proteins (MRJPs) belong to a wider family, nine members have been identified out with molecular weight ranging from 49–87 kDa. The proteins MRJP1, MRJP2, MRJP3, MRJP4, and MRJP5 represent ~82% of the total proteins present in RJ (192, 238, 239). MRJP1 has a molecular weight of 57 kDa as a monomer or 350 kDa as a hexamer. MRJP3 is a glycoprotein (60–70 kDa) (195) that has been isolated from RJ. MRJP1–8 have been characterized by cloning and sequencing their respective cDNAs (240). Royalisin is a 5.5-kDa antibacterial peptide isolated from the RJ and the antimicrobial activity against fungi, gram-negative and gram-positive bacteria have been revealed (234).

**Table 5:** Purification and identification of the peptides from bee products.

**Bee products**	**Protein/peptide**	**Molecular weight (kDa)**	**Separation/purification**	**Identification/analysis**	**References**
RJ	MRJP1 [A. mellifera MRJP (AmMRJP)] AmMRJPs 1–7		The MagneHis™ Protein Purification System (Promega, Madison, WI, USA)	SDS-PAGE and western blot analysis	(19, 201)
RJ (China)	MRJP1–3	228 kDa MRJP1 oligomer 1; 408 kDa MRJP1 oligomer 2 a; 639 kDa MRJP1 oligomer 3	Hydrophobic interaction chromatography; Ion-exchange chromatography, Size exclusion chromatography	Native-PAGE (SDS-PAGE) LC-MS/MS (SRCD) spectroscopy (SEC-HPLC)	(209)
BCP (Eastern Saudi Arabia)				LC3000 automatic amino acid analyzer ion-exchange chromatography	(202)
Black Dwarf Honey and BCP (Thailand)				ARACUS amino acid analyzer; high-performance liquid chromatography	(203)
RJ	MRJP7-9				(216)
RJ (China)	MRJP1	57 kDa monomer or 350 kDa hexamer			(217)
RJ	Royal actin MRJP1 oligomer	55 kDa 280 kDa			(218)
RJ	MRJP1 oligomer	350 kDa			(219)
RJ (Japan)	MRJP1 oligomer	420 kDa	Size-exclusion and anion-exchange HPLC	2-D blue native/SDS-PAGE. MALDI-TOF-MS SDS-PAGE Blue native-PAGE	(204)
RJ (Japan)	MRJP2: Royal jelly glycol-protein	55 kDa	Cosmosil 5CI8-AR Column	RP-HPLC MALDI-TOF MS	(220)
RJ (Japan)	MRJP2 and MRJP3 MRJP1	(52 kDa) (60–70 kDa) (290 kDa)		Size-exclusion HPLC, SDS–PAGE and 2-DE Superose 12 HPLC SDS–PAGE	(195)
RJ (Pisa)	Apalbumin 2 Apalbumin β		11 cm Immobiline DryStrip, pH 3–10	2-DE SDS-PAGE MALDI-TOF MS	(200)
RJ (Slovakia)	MRJP3 Apalbumin 3 Apalbumin γ	49–87 kDa	DEAE cellulose DE-52.	SDS-PAGE	(192)
RJ	RJP-1 RJP57-1				(221)
RJ (China)	MRJP5	80 kDa		2D-PAGE (MALDI-TOF/MS)	(214)
RJ (Japan)		55 kDa	Gel filtration on Sephadex G-100; Reverse-phase HPLC; Gel filtration on a GCL-90 column	SDS-PAGE	(196)
RJ (China)	MRJP1, MRJP2, and MRJP3	48.6 kDa 45.06 kDa 46.27 kDa	Extracted and separated using two-dimensional electrophoresis (2-DE)	(SDS-PAGE) (MALDI-TOF/MS) (Bruker Daltonics) LC-Chip/ESI-QTOF-MS	(80)
BV (Egypt)	Melittin Apamin MCD peptide Adolapin Minimine Procamine A, B Secarpin Tertiapin Melittin F	2,840 2,036 2,588 11,500 6,000 500 2,600 2,000 2,840			(222)
BV (French)	Apim6 MCD	(ca. 8,000)	Size exclusion chromatography and HPLC;	SDS-PAGE	(212)
	Cardiopep	(1940)			(223)
	Icarapin	(24,387)		SDS–PAGE	(213)
	MRJP8, MRJP9	(45,100), (46,300)	Nickel-chelating affinity matrix [nitrilotriacetic acid-agarose Qiagen, Hilden, Germany];	SDS-PAGE Waters Micromass QTOF2 mass spectrometer immobilized onto nitrocellulose membranes	(211)
BV (South Korea)	Melittin		Stepped-gradient open column (ODS-A; 120°A, 150 mesh) C18-5E YMC packed column;	HPLC	(100)
BV	Epitope-based peptide				(224)
BV (Korea) (Egypt) (Brazil)	Melittin and isoforms		Reversed-phase liquid chromatography using a Proeminene UPLC; Shim-pack VP-ODS C18 column	LCMS system using ESI-QTOF	(225–227)
	Apamin				(228)
	Adolapin		Gel filtration and chromatography on CM cellulose Sephadex GSO fine column	SDS	(10)
BV (South Korea) (USA)	PLA2 (Apim1)		PTFE membrane filter Pellicon 3 devices with Ultracel-10 kDa membrane; Reversed-phase (RP)-HPLC system on a C18 column	(HPLC)	(229–231)
BCP (Al-Ahsa eastern Saudi Arabia) (Australia)			Ion exchange chromatography with the utilization of Automatic Amino Acid Analyzer LC 3000		(205, 206)
BCP (China)				2-DE MALDI-TOF/MS	(198)
BCP (*Apis mellifera*) (Thailand)			0.65, 3, 5, and 10 kDa molecular weight cut-off Pellicon XL filter membranes (Merck); filtered through 0.45 mm filters; RP-HPLC system;	Quadruple time-of-flight mass spectrometry (Q-TOF-MS) Liquid chromatography-mass spectrometer (LC-MS)	(199)
BCP (Bulgaria)				SDS-polyacrylamide gel electrophoresis.	(207)
BCP (China)			Ultra-filtration and purification using preparative high-performance liquid chromatography		(197)
BCP (Gorgan)			size-exclusion chromatography (SEC). A Sephadex G-25 column	Agilent 1100 HPLC system TOF MS	(232)
RJ (China)			RP-HPLC35 × 50 mm low-pressure glass column 250 × 20 mm, S-10 μm, 12 nm C18 column (ODS-A, YMC, Japan)	HPLC system SDS-PAGE	(233)
RJ (recombinant royalisin)	Royalisin	5.5-kDa			(234)
RJ (Japan)			Membrane ultrafiltration, anion-exchange chromatography, gel filtration chromatography, and reverse-phase high-performance liquid chromatography	ESI-MS RP-HPLC (API-ESI)	(235)
BCP (China)	Polypeptide-2	42,388 Da	Ion-exchange column and gel filtration chromatography	MALDI-TOF-MS, LC-MS immunological histological chemistry transmission electron microscope SDS-PAGE	(215)

*RJ, Royal Jelly; BCP, Bee Collected Pollen; BV, Bee Vonom; BB, Bee Bread; SEC, Size-Exclusion Chromatography; SDS-PAGE, Sodium Dodecyl Sulfate Polyacrylamide Gel Electrophoresis; LC-MS/MS, Liquid Chromatography-Mass Spectrometry/Mass Spectrometry; Q-TOF-MS, Quadruple Time-Of-Flight Mass Spectrometry; SDS, Sodium Dodecyl Sulfate; RP-HPLC, Reverse Phase-High Performance Liquid Chromatography; SRCD, Synchrotron Radiation Circular Dichroism; SEC-HPLC, Steric Exclusion Chromatography High Performance Liquid Chromatography; LC-DAD-ESI, Liquid Chromatography Diode Array Detection Electrospray Ionization Tandem Mass Spectrometry; MALDI-TOF-MS, Matrix-Assisted Laser Desorption/Ionization Time of Flight Mass Spectrometry; 2-DE, Two-dimensional gel electrophoresis; LC-Chip/ESI-QTOF-MS, Liquid Chromatography-Chip/ Electrospray Ionization-Quadruple Time-of-Flight Mass Spectrometry*.

**Table 6:** Peptides sequence and bioactivity from bee products.

**Bee products**	**Peptides**	**Sequence and structure**	**Bioactivity**	**References**
RJ (Japan)	pisimin 5 kDa	KTSISVKGESNVDVVSQINS		(204)
RJ (Slovakia)	MRJP1 (55 kDa)	NILRGESLNKS		(192)
	MRJP2 49 kDa	AIVRENSPRNLEK		
	MRJP3 60–70 kDa	AAVNHQ (R/K)KSANNLAHS		
	MRJP5 77, 87 kDa	VTV (R/N)E (N/Q)SPR		
BCP (Thailand)	H2	VLAKNAPP RLNTAEAGH	NO scavenging activity IC_50_: 3.55 ± 0.09 mg/mL	(199)
	H3	VTAHSATVLPR KNKKWPAEAAH KLRSRNLLHPT TNRLLSGHSAKKH	1.57 ± 0.11	
	H4	TPVPWEAPRLN	5.64 ± 0.28	
BCP (China)		HPVTGL	ACE inhibitory activity IC_50_ of 51 μg/mL	(197)
RJ (Japan)	F1-1	IA		(235)
	F1-2	LT		
	F1-3	ATA		
	F1-4	AT		
	F1-5	PL		
	F1-6	PL		
	F1-7	LP		
	F1-8	LASTP		
	F4-1	TGG		
	F4-6	GIPHA		
	F4-11	IGIPHA		
	F2-1	LAA		
	F2-2	GVPSS		
	F2-3	AL		
	F2-4	LPHVP		
	F2-5	ALPHVP		
	F5-1	SAG		
	F5-3	TA		
	F6-1	TGGA		
	F6-2	PAA		
	F6-4	TAT		
	F5-12	GIPHA		
	F5-13	TAGH		
	F5-15	TT		
	F5-7	VATGG		
	F5-9	IAGGS		
	F5-10	IAGG		
	Fd-1	LL		
	Fd-2	HGT		
RJ (synthesis)	RJ I RJ II RJ III RJ IC RJ IIC RJ IIIC RJ I N RJ IIN RJ IIIN TA TB	PFKISIHL TPFKISIHL EPFKISIHL PFKISIHLGGY TPFKISIHLGGY EPFKISIHLGGY YGGPFKISIHL YGGTPFKISIHL YGGEPFKISIHL FLPLIGRVLSGIL LLPI VGNLLKSLL		(236)

*ACE, Angiotensin-I Converting Enzyme; IC_50_, half maximal Inhibitory Concentration; BCP, Bee Collected Pollen; RJ, Royal Jelly*.

Proteins are the second largest amount of nutritional component in BCP and fulfill the nutritional requirements of a honeybee. The peptides from bee products also possess various bioactivities. A novel angiotensin-I converting enzyme (ACE) inhibitory peptide was separated using UF and purified using preparative HPLC, which was derived from rape BCP protein hydrolysate (197). Seven peptide sequences were identified out by the quadrupole time-of-flight mass spectrometry, the three peptide displayed potent anti-inflammatory activity (199). The protein and amino acid concentrations in BCP were variable depending on their botanical origin. Five dominant amino acids (aspartic acid, glycine, glutamic acid, leucine, and alanine) were found in the tested BCP. They constituted ~58.56, 56.67, 58.80, 58.98, and 58.79% of the total quantified amino acids from alfalfa, date palm, summer squash, and rape, respectively (205). A novel protein with 42,388 Da from oilseed rape (*Brassica napus L*.), contained 17 amino acids (215).

BV is a liquid mixture that contains 88% water and only 0.1 g dry weight of a complex mixture of enzymes, peptides, and non-peptide components in one drop (241). Among them, peptides are the main components in BV (242). Approximately 48–50% of dry venom were the small proteins and peptides. Melittin and PLA2 (229) are the two most abundant compounds in BV. Melittin (a 26-amino acid peptide) is the main biologically active component in BV, which plays an important role in inducing reactions associated with bee stings (100) and it. Melittin and isoforms were also identified using the LC-MS ESI-QTOF system (225–227, 243–246), SDS-PAGE (213), and HPLC (100, 212).

#### Phenolic and Flavonoid Compounds

Honey, propolis, BCP, BB, RJ, beeswax, and BV are natural products that have been used for medicine since the ancient times (247).

Phenolic character substances that express the ability to scavenge free radicals, are primarily responsible for the antioxidant capacity of bee products (248). They comprise two main groups of compounds: flavonoids and phenolic acids (249).

The extraction procedure for the phenolics and flavonoids was primarily *via* solvent extraction using alcohol (methanol or ethanol) and water mixture (76, 250–253); cyclohexane, dichloromethane, butanol, and water (254); petroleum ether, n-butanol, ethyl acetate, and water (255); and acidified water (256). As for the phenolic and flavonoid contents in bee products, the Folin-Ciocalteu method (57, 254, 257–259) has been predominantly employed. The TFC of the BCP extracts was established (UV-Vis) in most cases using the aluminum trichloride colorimetric assay at 510 nm (253, 260) or aluminum nitrate at 415 nm (57, 258).

The characterization of phenolic compounds has been performed using different methods, especially HPLC (261), and the presence of these compounds in stingless bee honey has been evaluated by matching the retention time of the standard. HPLC with different detectors was also used to identify the phenolic compounds: HPLC with diode array detection (262), and semi-preparative HPLC system with a diode array detector (DAD) (263). High-performance liquid chromatography-mass spectrometry (HPLC-MASS) (264), liquid chromatography with diode array detection coupled with electrospray ionization (ESI) tandem mass spectrometry (LC-DAD-ESI) (265), ultra-high-performance liquid chromatography-diode array detection- mass spectrometry/mass spectrometry (UHPLC-DAD-MS/MS) (266), and HPLC-MASS methods have been developed for the characterization of secondary metabolites present in the methanol extract of BCP (267). UHPLC-DAD-ESI-MS, UHPLC-ESI-QTOF, HPLC-PDA, SPME-GC/MS, UHPLC-ESI-QTOF, and related techniques have been widely used to detect many active ingredients (268). The chemical composition of raw propolis was determined using GC-MS (269). Recently, high-speed counter-current chromatography has been used to separate tyrosinase inhibitors from camellia pollen (270, 271). Furthermore, using high-speed countercurrent chromatography and preparative HPLC, nuclear magnetic resonance and mass spectrometry are also used to identified the tyrosinase inhibitor compound as caffeine (272).

### Summary

- Drying techniques are essential for the storage of the bee products;
- Traditional drying methods include freezing, solar drying and in a hot air chamber. Hot air drying has a reasonably shorter processing time, the most adequate temperature for BCP drying is at 60°C, but hot air drying has an effect on the BCP quality and organoleptic characteristics, physicochemical properties, morphological structure; FD is considered the most gentle drying method, with fast rehydration rates and high rehydration capacity;
- The freezing and FD methods are used to preserve RJ, however some researchers pointed that FD RJ is highly susceptible to Maillard reaction (MR) and cannot meet the product quality and stability requirements;
- Innovative drying methods like IR, thin-layer IR, microwave, microwave-assisted vacuum drying (MW-VD), low temperature high velocity (LTHV)-assisted fluidized bed drying have been applied in the BCP drying;
- Several biotechnological solutions: fermentation with the yeast and enzyme hydrolysis; physical processing technology: ultrasonication and high shear (US-HS) technique, ultrasonic temperature difference, ultrasonic and ball-milling treatment, supercritical carbon dioxide (CO_2_) techniques have been used in the wall breaking;

## Processing Techniques Applied in the Bee Products Quality Control

### Assessment of the Bee Products

Among all the methods, morphometric analysis for morphology or surface texture and molecular structural analysis were the two aspects mainly for the differentiation or identification of compounds from bee products. The assessment methods applied in the bee products are shown in Table 7.

**Table 7:** Assessment methods for the bee products.

**Bee products**	**Techniques**	**Functions**	**References**
RJ (Turkey)	FTIR	♢ Prediction of the royal jelly content in hive products	(273)
BB (Ukraine)	Microscopic (SEM) and spectroscopic FT Raman and Vis, FT-MIR ATR, FT-NIR	♢ Discrimination of bee bread samples Morphometric analysis	(126)
BCP (Turkey)	SEM	♢ Morphological analysis	(148)
Beeswax (Slovakia)	FTIR Differential scanning calorimetry		(274)
BCP (China)	SEM, TEM, Particle size distribution	♢ Morphological observation	(162)
Rape BCP (China)	SEM		(156)
Honey (China)	MALDI-MS	♢ Detection of honey adulteration based on oligosaccharide and polysaccharide profiles	(134)
BCP (Turkey)	HP-TLC HPLC	♢ Fingerprinting of the phenolic compounds	(275)
BCP, propolis (Turkey)	GC-MS, SEM	♢ Analysis of the chemical composition ♢ Evaluation of the shape and size	(269)

*RJ, Royal Jelly; BB, Bee Bread; BCP, Bee Collected Pollen; SEM, Scanning Electron Microscope; GC-MS, Gas Chromatography-Mass Spectrometer; HPLC, High Performance Liquid Chromatography; HP-TLC, High Performance Thin-Layer Chromatography; MALDI-MS, Matrix-assisted laser desorption/ionization mass spectrometry; TEM, Transmission Electron Microscope; FT-MIR, Fourier Transform micro infrared spectroscopy, FT-NIR, Fourier Transform Near infrared spectroscopy; FTIR, Fourier Transform infrared spectroscopy*.

Microscopic (SEM) and spectroscopic methods (FT Raman; FT-MIR-ATR; FT-NIR; and Vis) were used to identify pollen grains and interpret the specific contribution of the main chemical constituents (126). SEM usually analyzes the morphometry and surface of the BCP (276) and the morphological changes after treatment (148). GC and GC-MS techniques were used to identify and confirm free amino acids in RJ (277) or the fatty acids in the bee products (112). Quick, easy, and non-destructive tools are used to ensure the quality and efficacy of PP and CP for clinical therapy (273, 278). FT-IR and 2D-IR spectroscopy were used to identify the Cattail pollen (CP) and pine pollen (PP) from BCP. In addition, differential scanning calorimetry (DSC) and FTIR spectroscopy were used to study the influence of aging conditions (274) and verify the authenticity of the beeswax (279). Matrix-assisted laser desorption/ionization mass spectrometry (MALDI-MS) was used to detect honey adulteration based on oligosaccharide and polysaccharide profiles (134).

Recently, omics technologies have been applied in honeybee product research to assess authentication, adulteration detection, quality, safety, and traceability of bee products, bioactivity (antimicrobial and antioxidant), microbiome characterization, and human health effects (280).

Electrochemical techniques (sensors and biosensors) are the predominant methodology that can be applied for the quantification of individual or total phenolic compounds, either in standard solutions or in real matrices (e.g., plants, fruits, and beverages) and their capability for assessing antioxidant activity/capacity (281). An electrochemical sensing platform based on a silver ion-cross-linked hydrogel for the evaluation of three different bee products has also been proposed (39). Electrochemical techniques have been acted as essential analytical tools for the characterization especial the antioxidant properties of the honey and propolis samples in an early stage (281).

### Bee Products Quality Control (Techniques and Standard)

#### Water Content and Microbial Quality Control

The moisture content and microbiological safety are the two key parameters to control to ensure the quality of bee products, especially for BCP and BB. Therefore, some standards or technologies are emerging at the same time. The presence of microorganisms in honey is an important indicator of quality. The drying out process for the bee product should ensure that the temperature lower than 42°C, with a water content <6% (282, 283). Minimal requirements for dried BCP have been established in Brazil: max. 4%; Switzerland and Poland: max. 6%; Uruguay: max. 8%; Bulgaria: max. 10% (283). The bacterial load should be control within legal hygienic limits (68). In addition, destruction of bacteria using chemical fumigants (284) or irradiation, ozone treatments (285) is not necessary and leads to toxic residues, but can control the microbiological quality (285). The European Union make the standard for microbiological quality follows AOAC methods and levels (283). While the major taxon needs to be not <80% (283).

#### Contaminant Control

BCP is least influenced by contaminants from beekeeping. The main contaminants in BCP are the heavy metals (286) and pesticides (287) originating from the environment and agricultural practices. BCP should gather in the areas at least 3 km from the contamination sources for control the quality. Requirements for heavy metal content of BCP loads are no more than: Cd; 1 mg/10 kg, Pb; 5 mg/10 kg, As; mg/10 kg, and Hg; 0.3 mg/10 kg (283).

The residual levels of three chemicals (i.e., imidacloprid, thiamethoxam, and chlorpyrifos) were higher in pollen than in bee bread (288). BCP from genetically modified organisms (GMOs) have a compulsory requirement (Regulation EC 1829/2003) to label products where the GMO content exceeds 1%, and the requirement could also be applied to BCP and honey (283). The dominant chemicals were the herbicides atrazine and trifluralin, followed by the insecticide chlorpyrifos thiamethoxam found only in one honey sample (289, 290).

#### Others

Detecting the compounds in bee products is a good strategy to compare samples from different regions and ensure the quality of bee products. The analysis of polysaccharides is a simple and efficient tool for honey quality control and detection of product adulteration (174). HPLC in gradient mode coupled with photodiode array detection (PAD) method remains for assay the most relevant propolis components. APCI-IT-MS represents a valuable alternative analysis method to obtain typical fingerprints of propolis and can be reliable identify a large number of propolis components (291). A suitable headspace solid-phase micro extraction (HS-SPME) techniques act as new and reliable tools were applied to determine the fingerprint of raw propolis samples from different Italian regions, thus providing the complete chemical characterization of this bioactive material (292). Raman spectroscopy combined with partial least squares-linear discriminant analysis as a potential technique have applied to detect the adulterants (high-fructose corn syrup and maltose syrup) in honey (293).

Moisture, HMF (Hydroxymethyl furfural), free acid, diastase are the composition and quality criteria used routinely for the international honey trade (135, 294). Knowledge of the physical or physicochemical properties of bee products is important for monitoring the quality of honey. The hot wire and dynamic plane source methods showed that both methods are suitable for the identification of the bee products thermal parameters (295). Multi-component analysis coupled with modern statistical data evaluation techniques seems to be a promising approach for authenticating honeys.

The nutrient content of the pollen also changes with storage. Thermophysical parameters are substantially correlated with the quality of bee products. Knowledge about the bee products thermophysical characteristics during thermal manipulation can improve the technological processing and storage (295). The color of the honey determined its value for marketing and its end-use. Sensory analysis can also help to determine the botanical origin of the honey (296).

### Summary

Microscopic (SEM) and spectroscopic methods (FT Raman; FT-MIR-ATR; FT-NIR; and Vis) were used to identify pollen grains. modern techniques like omics technologies and electrochemical techniques (sensors and biosensors) are the predominant methodology to assess authentication, adulteration detection, quality, safety, and traceability of bee products.

## Conclusions and Future Prospects

Hive products and the apitherapy have a long history dating back to the old times, which being used in phytotherapy and diet due to their powerful healing properties. Bee products as natural medicines are gaining prominence for the constituent bioactive compounds possess beneficial health properties and are widely used in food, medicines, and cosmetics. Convenient and efficient machines have been designed, and proper processing techniques have been applied based on the properties of the bee products.

Traditional drying techniques such as sun drying, hot air chamber, and FD are used to dehydrate the BCP and BB after harvesting. FD is considered the most effective technique, as it causes negligible damage to bee products; it has been used to dry bee pollen, bee bread, and preserve RJ. But some researchers pointed that RJ samples are not suitable for the FD techniques, for it may cause MR even at lower temperature for some days, the mechanism of that deserve more study. The RJ storage in industrial scales also need more emphasized in the future.

Innovative drying technologies are emerging these years to conserve the good quality of bee products. The water content for the BCP should control <6 or 4% in other country. The traditional drying temperature control at 60°C relative better to escape the water and deduce the microbial metabolism. MWD, IR radiation drying, thin-layer IR drying, vacuum drying alone and combined with other methods. MW-VD, LTHV-assisted fluidized bed drying, and IR heating-assisted fluidized bed drying are also widely used. These innovative drying methods can effectively escape the water, but have a marked effect on the quality characteristics, especially color. Beside the water escape effect, drying can also kill microorganisms of the bee products to some extent; irradiation, ozone treatments, and chemical fumigants can also control the microbes in bee products. New cold sterilization technologies should be applied in the future and combined with drying techniques.

Wall-breaking techniques special used for BCP and BB, biotechnological techniques (fermentation with yeast) and physical pretreatments have been used to increase the accessibility of nutrients for intestinal absorption. During fermentation, enzymes that can hydrolyze the wall of the BCPs are produced. The addition of different enzymes, such as pepsin, trypsin, and papain, can also effectively rupture the wall. Physical pretreatment like temperature difference, supercritical CO_2_, combined ultrasonic-ball-milling treatment, higher shear technique, and temperature difference with ultrasonication showed better wall-breaking efficiency. After fermentation or physical pretreatment, wall-breaking bee pollen yielded better active ingredients with excellent bioactivity. In the further study, wall-breaking technology should focus on the combine treatment, like the physical with fermentation and combine with ultrasound assist enzyme hydrolysis, which can produce the bee products for special patient (higher protein and peptide and less carbohydrate).

Extraction, isolation, and purification techniques are required for almost all bee products to obtain active ingredients. Carbohydrates, proteins, lipids, and fatty acids are the main compounds in BCP, BB, and RJ. Propolis is rich in phenols and flavonoids. The venom contains many peptides such as melittin or isoforms with excellent pharmacological effects. Recently, several active ingredients have been isolated. For the extraction technologies, related studies usually focus on the traditional methods, extraction using water, solvents or solvents with water, and Soxhlet extraction for obtaining oil or fatty acids by using a Soxtec HT 1043 system or solvent extraction alone. Different chromatography columns are widely used for the isolation of each compound, including DEAE cellulose DE-52. They include size-exclusion and anion-exchange HPLC, hydrophobic interaction chromatography, ion-exchange chromatography, size exclusion chromatography, gel-filtration chromatography, and affinity chromatography. Currently, procedures for isolation are tedious and only useful for analytical or preparative purposes in the laboratory, but unsuitable for large-scale processing; thus, new, facile, and efficient technologies should be applied for the extraction and isolation of active ingredients. High-speed countercurrent chromatography, which is a liquid-liquid separation technology, should be used more for isolating other small active ingredients, such as peptides in the future. Ultrasonication is an innovative technique that can be an efficient technique to assist in the extraction and isolation of lipids or other active ingredients.

For the different techniques, there is still scope for improvement in terms of the characteristics of the samples or the processing steps. Using combined physical processing techniques is a viable way to efficiently obtain more desirable bee products. In addition, to apply each technique on a pilot, or industrial scale is always a tough decision in the future.

## Author Contributions

XL conceived of the presented idea. XZ developed the theory and performed the computations. CG verified the analytical methods. HM encouraged XL to investigate pollen broken and supervised the findings of this work. All authors discussed the results and contributed to the final manuscript.

## Conflict of Interest

The authors declare that the research was conducted in the absence of any commercial or financial relationships that could be construed as a potential conflict of interest.

## Publisher's Note

All claims expressed in this article are solely those of the authors and do not necessarily represent those of their affiliated organizations, or those of the publisher, the editors and the reviewers. Any product that may be evaluated in this article, or claim that may be made by its manufacturer, is not guaranteed or endorsed by the publisher.
