# Supplementary material for: Processing Technologies for Bee Products: An Overview of Recent Developments and Perspectives
Source: Front Nutr. 2021 Nov 3;8:727181. doi: 10.3389/fnut.2021.727181 (PMC8595947; doi:10.3389/fnut.2021.727181)
Supplement: Supplementary file 1 [file Data_Sheet_1.docx]

**STable 1 The content of active ingredients from each bee products.**

| **Bee products** |  | **Biomacromolecule** | | | **Secondary metabolite** | | **Ref** |
| --- | --- | --- | --- | --- | --- | --- | --- |
|  |  | **Carbohydrates** | **Protein/peptides** | **Fats/ Fatty acids** | **Terpenes/carotenoids others** | **Polyphenols /flavonoids** |  |
| **Bee collection and brewing products** | BB  (Morocco) | Total free sugars (18 ± 1 g/100 g) | (19.96 ± 0.08 g/100 g) | Polyunsaturated  fatty acids  (64.7 ± 0.4%) |  |  | ([1](#_ENREF_1)) |
|  | BB  (Lithuania) |  |  |  |  | 196.3–221.6 mg RUE/100 g ;  78.8–156.7 mg RUE/100 g/ | ([2](#_ENREF_2)) |
|  | BB  (Portugal) | 58–78% | 14.1–21.8% | 3.6–16.8% |  |  | ([3](#_ENREF_3)) |
|  | BB  (not one kind) | (24–35%)  Monosaccharides 93.96%  Fructose (18.95%)  Glucose (11.54 %) | 21.93 and 22.12 % | 7.79 % |  |  | ([4](#_ENREF_4)) |
|  | BCP/BB  (Portuguese/  Japan) | 24–60 %-24–35% g | 20% protein | 3% Lipids |  |  | ([5](#_ENREF_5))  ([6](#_ENREF_6)) |
|  | BCP/BB  (Turkey) |  |  |  |  | 8.26 ± 0.29943.42 ± 0.779 mg GAE/g;  1.81 ± 0.040–4.44 ± 0.125 mg QE/g; | ([7](#_ENREF_7)) |
|  | BCP  (Lithuania) |  |  |  |  | 331.4–550.4 mg RUE/100 g ;  108.2–468.5 mg RUE/100 g | ([2](#_ENREF_2)) |
|  | Honey  (Germany &  Urbana) | Fructose (38%) and glucose (31%) |  |  |  |  | ([8](#_ENREF_8)) |
|  | BCP  (Białystok) | Carbohydrates (13–55%) are primarily fructose and glucose | Proteins and amino acids (10–40%) | Fats (1–20%) |  |  | ([9](#_ENREF_9)) |
|  | BCP  (Brazilian) | 68.10% and 75.51% | 19.44% and 23.80% | 2.66–7.34% |  |  | ([10](#_ENREF_10))  ([11](#_ENREF_11))  ([12](#_ENREF_12))  ([13](#_ENREF_13)) |
|  | BCP (Colombian) | Fructose (19.5 ± 0.9)  Glucose (13.6 ± 2.4)  Sucrose (6.7 ± 2.0) | 23.8 ± 3.2 g | 6.90 ± 3.5 g |  |  | ([13](#_ENREF_13)) |
|  | Honey  (Lithuania) |  |  |  |  | 2.95–10.18 mg RUE/10 g ;  0.28–5.22 mg RUE/10 g | ([2](#_ENREF_2)) |
|  | BCP  (Brazilian) | Glucose 8.77–19.12 %  Fructose14.45–20.23 % | 17.58–21.91% | 6.14–9.2 % |  |  | ([10](#_ENREF_10)) |
|  | BCP  (not mention) |  |  | 1–13 % |  |  | ([14](#_ENREF_14)) |
|  | BCP  (not mention) | 18.50–84.25%  reducing sugars 13–55% | Proteins and essential  amino acids (5–60%) | (0.15–31.26)% USF and SF |  | total phenolic content is  (0.69–213.20 mg GAE/g) | ([15](#_ENREF_15))  ([16](#_ENREF_16)) |
|  | BCP  (Scotland) | 13–55% | Protein and amino acids (10–40) % | Lipids (1–13) % |  |  | ([17](#_ENREF_17))  ([18](#_ENREF_18))  ([4](#_ENREF_4))  ([1](#_ENREF_1)) |
|  | Honey  (not mention) | 95–97% | 0.041% |  |  |  | ([19](#_ENREF_19))  ([20](#_ENREF_20)) |
|  | Honey  (not mention) | 82.4%  31% glucose, 38.5% fructose, 12.9% other sugars | 0.5% |  |  |  | ([21](#_ENREF_21))  ([22](#_ENREF_22)) |
|  | Honey  (not mention) | 82.40  Glucose 35.75, Fructose 40.94, Sucrose 0.89,  Maltose 1.44, Galactose 3.10 | 0.30 |  |  |  | ([23](#_ENREF_23)) |
|  | BCP  (Brazil) |  | 10.6–33.9 % | 3.2–8.3 % |  | 5.6–29.7 mg GAE/g  0.3–19.0 mg QE/g | ([24](#_ENREF_24)) |
|  | BCP  (Serbia) | 64.42–81.84% | 14.81–27.25% | 1.31–6.78% |  |  | ([12](#_ENREF_12)) |
|  | BCP  (not one kind) | 10–40% | 5–30% | 1–5% |  |  | ([25](#_ENREF_25)) |
|  | BCP  (not one kind) | 54.22% (18.50–84.25%) carbohydrates 13.41 % ;  (2.77–28.49 %) glucose;  15.36% (4.9–33.48 %) fructose;  4.25 % (0.05–9.02 %) sucrose | 21.30% (4.50–40.70%) | 5.31% (0.41–13.50%) Lipids |  | (0.69–213.20 mg GAE/g) | ([15](#_ENREF_15)) |
|  | BCP  (Brazil) | Reducing sugars (up to 46%) | Proteins (up to 31%) | Lipids (up to 13%) |  | Up to 1.5 % | ([26](#_ENREF_26)) |
|  | BCP  (Slovenia) | 13.2–27.8 % for fructose and 10.6–28.5 g %for glucose. |  |  |  |  | ([27](#_ENREF_27)) |
|  | BCP  (not one kind) | 40–85% (W/W) of dry bee pollen | 14–30% (W/W) | 1–10% (W/W) |  |  | ([28](#_ENREF_28)) |
|  | BCP  (Turkey) | 80.24 ± 0.836 | 15.69 ± 0.810 | 2.17 ± 0.038 |  | 14.42 ± 0.60 | ([29](#_ENREF_29)) |
|  | BCP  (Malaysia) | 57.06 ± 2.09–58.89 ± 0.28 | 21.70 ± 0.08–23.33 ± 0.48 | 4.64 ± 0.04–5.95 ± 0.10 |  |  | ([30](#_ENREF_30)) |
|  | BCP  (Ukraine) |  | 15.30 ± 2.09–28.06 ± 2.78 |  |  |  | ([31](#_ENREF_31)) |
|  | BCP  (Colombia) |  | 23.1 ± 2.9 g | 3.4 ± 1.1 g |  | 3.2 ±1.0 mg Quercetin/g  8.9 ± 3.1 mg Gallic acid/g | ([32](#_ENREF_32)) |
|  | BCP  (India) |  | 57036.67 ± 14.83 (mg/mL)  60780 ± 21.86 (mg/mL)  33953.33 ± 4.83 (mg/mL) |  |  | 0.91 ± 0.04 GAE/100 gm  0.99 ± 0.02 GAE/100 gm  0.80 ± 0.03 GAE/100 gm | ([33](#_ENREF_33)) |
|  | BCP  (European)  (England) | (130 mg/g ± 63) | 629 mg/g ± 290 wet weight | 38 ± 2 mg/g |  |  | ([34](#_ENREF_34)) |
|  | BCP  (Morocco) | (28.46% ± 0.994) | (12.81% ± 0.167) | (2.31% ± 0.574) |  | 14.88 ± 0.98 mg GAE/g  1.67 ± 0.12 mg QE  (quercetin equivalents)/g | ([35](#_ENREF_35)) |
|  | BCP  (Malaysia) | Fructose (17‒23%) glucose (14‒16%) and sucrose (5‒6%) | Colombian (24%) and Italian (22%)  Spanish sample (14%) | Spanish and Colombian (6%) Italian (2.5%) | Carotenoids 57, 25 and 221 µg/g for Spain, Italy, and Colombia BCP |  | ([36](#_ENREF_36)) |
|  | BCP  (Brazilian) | 54.9–82.8 % | 7.9–32.2 % | 3.2–13.5 % |  | (6.5–29.2 mg GAE/g)  (0.3–17.5 mg QE/g) | ([37](#_ENREF_37)) |
|  | BCP  (Korea) |  | 17.2 ± 0.18–26.8 ± 0.07% | 3.1 ± 0.10–12.2 ± 0.04% |  |  | ([38](#_ENREF_38)) |
|  | BCP | 90% of total sugars  Fructose and glucose  average contents ranged from 15.53–33.48 %  13.59–27.69 % |  |  |  |  | ([39](#_ENREF_39)) |
|  | BCP  (Turkey) |  |  | 3.37 ± 0.02%–6.85 ± 0.02% | 24.11 ± 0.09–98.62 ± 0.02 Carotenoid  content (mg/g) | TPC: 434.17 ± 0.01–719.58 ± 0.01 % | ([40](#_ENREF_40)) |
|  | BCP  (Greece) |  |  |  |  | 15.2 ± 0.4 to 60.2 ± 2.0 (mg GAE/g extract)  6.0 ± 0.3 to 57.6 ± 2.0 (mg QE/g extract) | ([41](#_ENREF_41)) |
|  | BCP  (Turkey) |  |  |  |  | 3.9 and 9.2 mg GAE/g-  1.2–1.9 mg CAE/g | ([42](#_ENREF_42)) |
|  | Honey  (Brazil)  BCP  (Brazil) | 215 ± 33–301 ± 169 (mg/g) | 45 ± 18–99 ± 9 (mg/g) | 2 ± 0–6 ± 1 (%) |  | 32 ± 9–136 ± 32 (mg GAEq/100 g);  8 ± 2–55 ± 20 (mg QEq/100 g)  6.9–21 ± 2 (mg GAEq/g);  0.3–17 ± 5 (mg QEq/g); | ([43](#_ENREF_43)) |
|  | BCP  (Brazil) |  |  |  |  | 33.73–75.60 mg GAE/g  1.42–9.05 mg QE/g | ([44](#_ENREF_44)) |
|  | Propolis |  |  |  |  | 13–379 mg of quercetin equivalents (QE) per g  68–500 mg of caffeic acid equivalents (CAE) per g | ([45](#_ENREF_45)) |
|  | Propolis  (Lithuania) |  |  |  |  | 68.03–99.85 mg RUE/10 g  /3.24–14.39 mg RUE/10 g | ([2](#_ENREF_2)) |
|  | BCP  (India) |  |  |  |  | 15.50 ± 1.25–25.63 ± 1.42 mg GAE/g and 9.72 ± 0.28–15.61 ± 0.74 mg RE/g | ([46](#_ENREF_46)) |
|  | Maize BCP  (Malaysia) | carbohydrates (44.30 ± 3.73) % | (17.16 ± 3.13) % | (0.62 ± 0.06%) |  | 783.02 mg GAE/ 100 g and 1706.83 mg QE/100 g | ([47](#_ENREF_47)) |
| **Bee secretions** | BV |  | Melittin 52% of all apitoxin peptides |  |  |  | ([9](#_ENREF_9)) |
|  | BV  (not mention) |  | 0.1 g mixture of peptides, enzymes, and nonpeptide components |  |  |  | ([48](#_ENREF_48)) |
|  | BV  (not one kind) |  | Small proteins and peptides |  |  |  | ([49](#_ENREF_49)) |
|  | Beeswax  (not mention) | MUD 1: 6.4 ± 1.0  MUD 2: 0.5 ± 2.3 | MUD 1: 12.9 ± 0.2  MUD 2: 2.3 ± 0.1 | MUD 1:11.0 ± 0.5  MUD 2:46.1 ± 3.7 |  | MUD 1: 1435.66  MUD 2: 432.66 mg GAE q/100 g | ([50](#_ENREF_50)) |
|  | RJ  (not one kind) | Near 30% | 27–41% | 8–19% |  |  | ([51](#_ENREF_51)) |
|  | RJ  (not one kind) | 10–16% (w/w) | 9–18% (w/w) |  |  |  | ([52](#_ENREF_52)) |
|  | RJ | (16%) | Proteins and amino acids (12.5%) | 5% |  |  | ([53](#_ENREF_53)) |
|  | RJ  (Japan) | 15% | 18% | 3–6% |  |  | ([21](#_ENREF_21))  ([54](#_ENREF_54)) |
|  | RJ  (not one kind) | 7–18% w/w | 9–18% w/w | 3–8% w/w |  |  | ([55](#_ENREF_55))  ([56](#_ENREF_56)) |
|  | RJ  (Lithuania) |  |  |  |  | 16.44–23.14 mg RUE/10 g/  10.34–17.19 mg RUE/10 g | ([2](#_ENREF_2)) |
|  | RJ  (not one kind) | 7.5–16% |  |  | Wax (5–6) %;  steroids (3–4) %;  phospholipids (0.4–0.8) % | Phenolic acids (4–10) % | ([57](#_ENREF_57)) |
|  | RJ  (not one kind) |  |  | 7–18% |  |  | ([58](#_ENREF_58))  ([59](#_ENREF_59)) |
| **Bee ecological bodies and hives** | Bee pupae  (Scotland) | (20.34%)  total sugar (0.73%) | (46.21%) | (26.09%) |  |  | ([17](#_ENREF_17)) |
|  | Honeybee larvae  (Japan) |  |  |  |  | 51.44 ± 2.77 total phenolic content (mg GAE/g)  2.47 ± 0.23 TFC (mg RE/g) | ([60](#_ENREF_60)) |
|  | Honeybee larva  (Japan) | 30.3% | 50.1% | 13.5% |  |  | ([61](#_ENREF_61)) |
|  | Larvae  (Korea) | 46.1±1.73 | 35.3±2.09 | 14.5±0.15 |  |  | ([62](#_ENREF_62)) |
|  | Pupae  (Korea) | 34.3±0.24 | 45.9±0.63 | 16.0±0.24 |  |  |  |

**Footnotes: BB**=Bee Bread; **BCP**=Bee Collected Pollen; **BV**= Bee Venom; **RJ**=Royal Jelly; **RUE**= rutin equivalent; **GAE**= Gallic Acid Equivalents; **QE**= Quercetin Equivalents; **RE**= Rutin Equivalents; **TFC**= Total Flavonoid Content; **TPC**= Total Phenolic Content; **CAE**=Caffeic Acid Equivalents;

**STable 2 The characteristics of each drying method.**

| **Bee products** | **Drying technology** | **Conditions and achieve** | **Models and parameters** | **Advantages and disadvantages** | **Ref** |
| --- | --- | --- | --- | --- | --- |
| BCP  (Turkey) | Hot air chamber | - the highest sensory scores obtained at 40 °C - at 45, 50, 55, and 60 °C retained better quality | - | - A shorter processing time - Lower risk of microbial contamination - More effective - Better control - Applied in many industrial drying applications | ([63](#_ENREF_63)) |
| BCP  (Colombia.) | Solar drying | - The average temperature was 50 °C - air-flow speed of 2 m/s | - | - Generates energy savings Reduce the carbon footprint of the process | ([64](#_ENREF_64)) |
| BCP  (Colombia) | Hot air drying | - The most adequate temperature was at 60 °C - A marked increase in flavonoids, phenolics, and antioxidant activity - A loss of carotenoids Structure slight degradation | - | - modify the microstructure of the exine - Nutritional compounds would be more available | ([65](#_ENREF_65)) |
| BCP  (Russian) | Cyclic convective drying | - The rational circulation speed range 2.2–2.5 m/s; - air temperature 40–42 °C |  | - preserve the biologically active properties | ([66](#_ENREF_66)) |
| BCP  (Italy) | Freeze-drying (FD)  (using a lyophilizer Heto PowerDry  LL1500) | - condensation chamber temperature was −115 °C, - full vacuum. - treated for 270, 420, or 540 min, |  |  | ([67](#_ENREF_67)) |
| RJ  (Viet Nam) | FD | - optimal temperature was 20.58 °C, - optimal pressure was 0.411 mmHg - freeze time was 18.283 h - final product reached the minimum 6.32 kWh/kg, - The residual water content 4.19% under 4.5% (< 4.5%); - Loss of protein, carbohydrate, lipid, mineral salts, 10-HAD, vitamin B5, free fatty acids, - viscosity reached the minimum | - Using quadratic orthogonal experimental planning method   $y_{j}=b_{0}+\sum_{u=1}^{k} b_{u}X_{u} +\sum_{u\neq i;u=1}^{k} b_{\mathrm{ui}}X_{u}X_{i} + \sum_{u=1}^{k} b_{\mathrm{uu}}\left( X_{u}^{2} - \lambda\right)$  $X_{i}=\frac{\left( Z_{i}-Z_{i}^{0} \right)}{\Delta Z_{i}}; Z_{i}= X_{i.}\Delta Z_{i}+ Z_{i}^{0}$  $Z_{i}^{0}={(Z}_{i}^{\max} + Z_{i}^{\min}) /2 ;$  $\Delta Z_{i}=\left( Z_{i}^{max}-Z_{i}^{min} \right) /2$  $Z_{i}^{\min}\leq Z_{i}\leq Z_{i}^{\max} ; i=1 to 3$ |  | ([68](#_ENREF_68)) |
| RJ  (Italy) | FD | - At −50 °C for 24 h - Furoins content was higher in the freeze-dried RJ after both 6 and 12 months |  | - maintains the natural characteristics without damage or denature the thermolabile components | ([69](#_ENREF_69)) |
| RJ  (Viet Nam) | FD | - The optimal temperature of the FD chamber was 24.35 °C - The optimal pressure was 0.368 mmHg - The optimal time of the FD process was 19.225 h - After FD 3.51 under 4.5%, with the minimum value of the loss of nutrition | - Mathematical model of the residual water content   $y_{1}=f_{2}\left( X_{1}, X_{2} , X_{3} \right)=3.216-0.271X_{1}+0.116 X_{2}-0.643 X_{3}-0.111X_{1}X_{3}+0.139 X_{1}^{2}+0.118 X_{2}^{2}+0.237 X_{3}^{2}$   - Mathematical model of the total protein loss   $y_{2}=f_{1}\left( X_{1}, X_{2}, X_{3} \right)=2.245+0.274X_{1}+0.814 X_{3}+0.303 X_{1}^{2}+0.332 X_{2}^{2}+0.311 X_{3}^{2}$   - Mathematical model of the carbohydrate loss   $y_{3}=f_{1}\left( X_{1}, X_{2}, X_{3} \right)=1.978+0.225X_{1}+0.228 X_{2}+0.263X_{3}+0.144 X_{2}X_{3}+0.239X_{1}^{2}+0.162 X_{2}^{2}+0.169 X_{3}^{2}$   - Mathematical model of the loss of lipid   $y_{4}=f_{2}\left( X_{1}, X_{2}, X_{3} \right)=1.934+0.131X_{2}+0.404 X_{3}+0.417 X_{1}^{2}+0.186 X_{2}^{2}+0.237 X_{3}^{2}$ | - | ([70](#_ENREF_70)) |
| BCP  (Turkey) | Infrared radiation drying | - Sample treated at different power levels 50, 62, 74, and 88 W. - surface morphological changes - quality characteristics retained better at 50 W | - | - Decrease the drying time - with high-quality - high energy efficiency - uniform temperature in the dried product | ([71](#_ENREF_71)) |
| BCP  (Italy) | FD and microwave-assisted drying (MWD) | - condensation chamber temperature was −115 °C - At full vacuum. - FD treatment for 9 h, - The residual water content was 6.0%, - The absolute pressure was 50 mbar. - 150 W MW treatment for 30 min |  |  | ([72](#_ENREF_72)) |
| BCP  (Brazil) | Infrared heating-assisted fluidized bed dryer | - Conventional drying at 35, 45, and 55 °C - velocity equal to minimum fluidization velocity - radiation intensity at 100, 400, and 700 W/m^2^ without heating the air - 52% energy saving achieve using the IR heating-assisted fluidized bed dryer. | - Moisture diffusion model   $\text{XR=}\frac{X-X_{e}}{X_{0}-X_{e}}=\sum_{j=1}^{n} \frac{6}{{(j\pi)}^{2}} exp\left[ -\left( j\pi\right)^{2} \frac{D_{eff}}{R^{2}} t \right]$  XR: moisture ratio;  $SEC=\frac{(P_{IR}+P_{compr}+P_{heater})\cdot t}{m_{w}}$   - Kinetics of color   $BI=C_{0}+\alpha_{0}\cdot t$  $\frac{BI}{{BI}_{0}}=\exp(\alpha_{1}\cdot t)$ | - Prevents browning reactions - Avoids color degradation | ([73](#_ENREF_73)) |
| BB  (Russia) | Convection drying method, |  |  | - High energy intensity of the process | ([74](#_ENREF_74)) |
| BB  (Russia) | Vacuum method, |  |  | - High cost of equipment | ([74](#_ENREF_74)) |
| BB  (Russia) | convective and infrared drying |  | COMSOL Multiphysics software   - model of drying a porous body   $\frac{\partial c_{w}}{\partial t}+\nabla\cdot J_{w}+u\cdot\nabla c_{w}=R_{w}$  $J_{w}=-D_{w}\nabla c_{w}$  $D_{w}-diffusion coefficient, {{(m}^{2}\cdot s)}^{-1}$  $R_{w}-reaction rate, mol\cdot{(m^{3}\cdot s)}^{-1}$  $u-mass averaged velocity vector, m\cdot s$  $J_{w}-mass flux,mol\cdot{(m^{2}\cdot s)}^{-1}$ | - Reduce the processing drying time and energy intensity | ([74](#_ENREF_74)) |
| BCP  (Turkey) | MWD | - 300, 450, 600, and 900 W power - 900 W created considerable degradation, nearly 26–28%, of vitamin E |  |  | ([29](#_ENREF_29)) |
| BCP  (Italy) | MWD | - 50 mbar; MW power was 150 W; the exposure time was 30 min - The residual water content was 6.4%, 10.3%, and 8.2% (chestnut, willow, and ivy pollen) |  | - Minimum affect the content of the flavonoids, complex B vitamins, and/or unsaturated lipids | ([75](#_ENREF_75)) |
| BCP  (Turkey) | Microwave-assisted vacuum drying (MW-VD) | - 300, 450, 600, and 900 W power |  | - Higher reduction in antioxidant compounds (tocopherols) - No substantial change in total phenols and flavonoid content of fresh bee pollen | ([29](#_ENREF_29)) |
| BCP  (Turkey) | Hot-air drying (HAD) | - Air velocity of 0.54 m/s - provided the best preservation of vitamin C at 35 °C - An average loss ranging between 12.9 and 29.2% in vitamin E content |  | - Vitamin E, beta-carotene, and vitamin C loses during the heating | ([29](#_ENREF_29)) |
| BCP  (Turkey) | Vacuum drying (VD) | - 300, 500 mbar |  |  | ([29](#_ENREF_29)) |
| BCP  (Turkey) | FD | - Vacuum pressure of 0.1 mbar - Vitamin E, total phenolic content, and TEAC values were similar |  | - The nutrition did not alter after FD | ([29](#_ENREF_29)) |
| BCP  (Italy) | FD | - The temperature in the condensation chamber was −115 °C, - full vacuum - The residual water content was 6.0%, 6.3%, and 7.5%(chestnut, willow, and ivy pollen) |  | - minimum affect the content of the flavonoids, complex B vitamins, and/or unsaturated lipids | ([75](#_ENREF_75)) |
| BCP  (Turkey) | low temperature high velocity (LTHV)-assisted fluidized bed  drying | - 4, 10, 24, and 40 °C, ~40–50 humidity (%) and 6 m/s air velocity - The cooling and heating unit was controlled with a thermostatic sensor connected | - Logarithmic (Logarithmic (asymptotic)   $\mathrm{Logarithmic}\left( \mathrm{asymptotic} \right)yagcioglu et al.model MR=a\exp\left( -kt \right)+c$ | - Applied in the food industry - preserve the quality of the perishable and semi-dried food product at low temperature - minimize the lipid oxidation, - minimize the texture, color, protein, and sensory quality lost | ([76](#_ENREF_76)) |
| BCP  (Turkey) | Microwave drying | - 180, 360, 540, 720, and 900 W microwave power - Drying time was shortened by 94% when increased from 180 to 900 W | - The moisture contents of the BCP   $M_{t}=\frac{W_{t}-W_{s}}{W_{s}}$  $MR=\frac{M_{t}-M_{e}}{M_{0}-M_{e}}$  $MR=\frac{M_{t}}{M_{0}}$  $\frac{\partial M}{\partial t}=\left( D_{eff} \right) \frac{\partial^{2}M}{\partial x^{2}}$  $MR=\frac{8}{\pi^{2}}\sum_{n=0}^{\infty} \frac{1}{{(2n+1)}^{2}}exp(-\frac{{(2n+1)}^{2}\pi^{2}D_{eff}t}{{4L}^{2}})$  $MR=\frac{8}{\pi^{2}} exp(-\frac{\pi^{2}D_{eff}t}{{4L}^{2}})$  $\ln MR=\ln\frac{8}{\pi^{2}}-\frac{\pi^{2}D_{eff}t}{4L^{2}}$  $Slope=\frac{\pi^{2}D_{eff}}{4L^{2}}$  Arrhenius type equation$D_{eff}=D_{0} exp(-\frac{E_{a}m}{P})$  $\ln\left( D_{eff} \right)=\ln\left( D_{0} \right)-\frac{E_{a}m}{P}$ | - Microwave energy speeds up the drying process - energy saving, and drying with smaller dryer sizes - Shorten drying time, - retain nutritional value - improve the final quality of dried products | ([77](#_ENREF_77)) |
| BCP  (Turkey) | Hot-air (HAD) and vacuum (VD) | - (300, 500 mbar) drying at 35, 50 and 65 °C, - A higher retention of enzyme activity, was observed at 50 °C |  |  | ([78](#_ENREF_78)) |
| BCP  (Italy) | Classic hot air drying (HAD) | - A temperature of 32 °C for 24 h in the NTW100 cool-air dryer - Residual water was 7%, |  | - produce Maillard’s compounds after drying | ([75](#_ENREF_75)) |
| BCP  (Turkey) | MWD and microwave-assisted vacuum drying (MW-VD) | - (500, 675 mbar) at 300, 450, 600, and 900 W power - A greater reduction was observed in DN of samples at 600 and 900 W - Proline and HMF content were affected by the treatment power |  |  | ([78](#_ENREF_78)) |
| BCP  (Turkey) | FD |  |  | - preserve the bioactive compounds and biological properties | ([78](#_ENREF_78)) |
| BCP  (Italy) | MW-VD | - 50 mbar for some minutes |  | - Reduces the water content without thermally deteriorating important bioactive compounds | ([79](#_ENREF_79)) |

**Footnotes: BCP**=Bee Collected Pollen; **BB**=Bee Bread; **RJ**= Royal Jelly; **FD**=Freeze-drying; **HAD**=Hot-air drying; **MW-VD**=Microwave-assisted Vacuum Drying; **VD**=Vacuum Drying; **LTHV**=Low Temperature High Velocity; **MWD**=Microwave-assisted Drying; **TEAC**= Trolox Equivalent Antioxidant Capacity; **HMF**=Hydroxymethylfurfural; **10-HAD**=10-hydroxy-decenouc acid; **IR**= Infrared

Ghosh S, Jung C, Meyer-Rochow VB. Nutritional value and chemical composition of larvae, pupae, and adults of worker honey bee, Apis mellifera ligustica as a sustainable food source. J Asia Pacific Entomol. (2016) 19:487–95. doi: 10.1016/j.aspen.2016.03.008

Reference

1. Bakour M, Fernandes Â, Barros L, Sokovic M, Ferreira ICFR, Badiaa l. Bee bread as a functional product: Chemical composition and bioactive properties. *Lwt*. (2019) 109:276-82. doi:10.1016/j.lwt.2019.02.008

2. Adaskeviciute V, Kaskoniene V, Kaskonas P, Barcauskaite K, Maruska A. Comparison of Physicochemical Properties of Bee Pollen with Other Bee Products. *Biomolecules*. (2019) 9:819. doi:10.3390/biom9120819

3. Tomás A, Falcão SI, Russo-Almeida P, Vilas-Boas M. Potentialities of beebread as a food supplement and source of nutraceuticals: Botanical origin, nutritional composition and antioxidant activity. *J Apicult Re*s. (2017) 56:219-30. Doi: 10.1080/00218839.2017.1294526

4. Khalifa SAM, Elashal M, Kieliszek M, Ghazala NE, Farag MA, Saeed A, et al. Recent insights into chemical and pharmacological studies of bee bread. *Trends Food Sci Tech*. (2020) 97:300-16. doi:10.1016/j.tifs.2019.08.021

5. Estevinho LM, Rodrigues S, Pereira AP, Feás X. Portuguese bee pollen: palynological study, nutritional and microbiological evaluation. *Int J Food Sci Tech.* (2012) 47:429-35. Doi: 10.1111/j.1365-2621.2011.02859.x

6. Nagai T, Nagashima T, Myoda T, Inoue R. Preparation and functional properties of extracts from bee bread. *Nahrung.* (2004) 48:226-9. Doi: 10.1002/food.200300421

7. Mayda N, Özkök A, Ecem Bayram N, Gerçek YC, Sorkun K. Bee bread and bee pollen of different plant sources: determination of phenolic content, antioxidant activity, fatty acid and element profiles. *J Food Meas Charact.* (2020) 14:1795-809. doi:10.1007/s11694-020-00427-y

8. Nele GXHW, Nicki JE. Identification and Quantification of Antioxidant Components of Honeys from Various Floral Sources. *J Agric Food Chem.* (2002) 50:5870-7. doi:10.1021/jf0256135

9. Szabat Przemysław PJ, Szabat Marta, Boreński Grzegorz, Wójcik Magdalena, Milanowska Joanna. Apitherapy–the medical use of bee products. *J Edu, Health Sport.* (2019) 9:384-96.

10. Costa MCA, Morgano MA, Ferreira MMC, Milani RF. Analysis of bee pollen constituents from different Brazilian regions: Quantification by NIR spectroscopy and PLS regression. *Lwt*. (2017) 80:76-83. DOI: 10.1016/j.lwt.2017.02.003

11. Sari MF, Esen F, Tasdemir Y. Levels of polychlorinated biphenyls (PCBs) in honeybees and bee products and their evaluation with ambient air concentrations. *Atmos Environ.* (2021) 244:117903. Doi: 10.1016/j.atmosenv.2020.117903

12. Kostić AŽ, Barać MB, Stanojević SP, Milojković-Opsenica DM, Tešić ŽL, Šikoparija B, et al. Physicochemical composition and techno-functional properties of bee pollen collected in Serbia. LWT. (2015) 62:301-9. Doi:10.1016/j.lwt.2015.01.031

13. Evaluación de las propiedades fisicoquímicas y funcionales del pac. Evaluation of the physicochemical and functional properties of Colombian bee pollen. *RevMVZ Córdoba* .(2014) 19:4003-14. DOI: 10.21897/rmvz.120

14. Campos MGR BS, Almeida-Muradian LB, Szczesna YM, Frigerio C, Ferreira F. Pollen composition and standardisation of analytical methods. *J Apic Res Bee World.* (2008) 47(2):156-63.doi: 10.1080/00218839.2008.11101443

15. Thakur M, Nanda V. Composition and functionality of bee pollen: A review. *Trends Food Sci Tech*. (2020) 98:82-106. DOI: https://doi.org/10.1016/j.tifs.2020.02.001

16. Themelis T, Gotti R, Orlandini S, Gatti R. Quantitative amino acids profile of monofloral bee pollens by microwave hydrolysis and fluorimetric high performance liquid chromatography. *J pharmaceutica biomed* .(2019) 173:144-53. doi:10.1016/j.jpba.2019.05.031

17. Ryabov EV, Wood GR, Fannon JM, Moore JD, Bull JC, Chandler D, et al. A virulent strain of deformed wing virus (DWV) of honeybees (Apis mellifera) prevails after Varroa destructor-mediated, or in vitro, transmission. *PLoS Pathog*. (2014) 10:e1004230. doi:10.1371/journal.ppat.1004230

18. Campos MGR, Frigerio C, Lopes J, Bogdanov S. What is the future of Bee-Pollen? *J ApiProd and ApiMed Sci*. (2010) 2:131-44. DOI 10.3896/IBRA.4.02.4.01

19. Samarghandian S, Farkhondeh T, Samini F. Honey and Health: A Review of Recent Clinical Research. *Pharmacognosy Res*. (2017) 9:121-7. doi: [10.4103/0974-8490.204647](https://dx.doi.org/10.4103%2F0974-8490.204647)

20. El-Soud NHA. Honey between Traditional Uses and Recent Medicine. *Macedonian J Med Sci*. (2012) 5:205-14. doi:10.3889/mjms.1857-5773.2012.0213

21. Pasupuleti VR, Sammugam L, Ramesh N, Gan SH. Honey, Propolis, and Royal Jelly: A Comprehensive Review of Their Biological Actions and Health Benefits. *Oxid med cell longev*. (2017) (2017) 7:1-21. doi:10.1155/2017/1259510

22. Federico Ferreres CG-V, Francisco Tomas-Lorente and Francisco A Tomas-Barberan. Hesperetin: A Marker of the Floral Origin of Citrus Honey. *J Sci Food Agric*.(1993) 61:121-3. doi:10.1002/jsfa.2740610119

23. Mijanur Rahman M, Gan SH, Khalil MI. Neurological effects of honey: current and future prospects. *Evid-based compl alt.* (2014) 2014:1-13. doi:10.1155/2014/958721

24. De-Melo AAM, Estevinho LM, Moreira MM, Delerue-Matos C, Freitas AdSd, Barth OM, et al. Phenolic profile by HPLC-MS, biological potential, and nutritional value of a promising food: Monofloral bee pollen. *J Food Biochem*. (2018) 42(5):e12536. doi:10.1111/jfbc.12536

25. Linskens HF, Jorde W. Pollen as food and medicine--A Review. *Econ Bot.* (1997) 51:78-87. doi:10.2307/4255920

26. Almeida-Muradian LB, Pamplona LC, Coimbra Sl, Barth OM. Chemical composition and botanical evaluation of dried bee pollen pellets. *J Food Compos Anal*. (2005) 18:105-11. doi:10.1016/j.jfca.2003.10.008

27. Bertoncelj J, Polak T, Pucihar T, Lilek N, Kandolf Borovšak A, Korošec M. Carbohydrate composition of Slovenian bee pollens. *Int J Food Sci Tech*. (2018) 53:1880-8. doi:10.1111/ijfs.13773

28. Li Q-Q, Wang K, Marcucci MC, Sawaya ACHF, Hu L, Xue X-F, et al. Nutrient-rich bee pollen: A treasure trove of active natural metabolites. *J Funct Foods*. (2018) 49:472-84. doi:10.1016/j.jff.2018.09.008

29. Kanar Y, Mazı BG. Effect of different drying methods on antioxidant characteristics of bee-pollen. *J Food Meas Charact*. (2019) 13:3376-86. doi:10.1007/s11694-019-00283-5

30. Mohammad SM, Mahmud-Ab-Rashid N-K, Zawawi N. Botanical Origin and Nutritional Values of Bee Bread of Stingless Bee (Heterotrigona itama) from Malaysia. *J Food Quality.* (2020) 2020:1-12.  doi:10.1155/2020/2845757

31. Bleha R, Shevtsova TS, Kružík V, Škorpilová T, Saloň I, Erban V, et al. Bee breads from two regions of Eastern Ukraine: composition, physical properties and biological activities. *Czech J Food Sci*. (2019) 37:9-20. doi:10.17221/201/2018-cjfs

32. Carlos M. Zuluaga JCS, Marta C. Quicazana. Chemical, Nutritional and Bioactive Characterization of Colombian Bee-Bread. *Chenical engineering transactions.* (2015) 43:175-80. doi: 10.3303/CET1543030

33. Bhargava VUEaHR. Chemical Analysis and Anti-Microbial Activity of Karnataka Bee Bread of Apis species. *World Applied Sciences Journal*. (2014) 32:379-85.

34. Donkersley P, Rhodes G, Pickup RW, Jones KC, Power EF, Wright GA, et al. Nutritional composition of honey bee food stores vary with floral composition. *Oecologia.* (2017) 185:749-61. doi:10.1007/s00442-017-3968-3

35. Bakour M, Al-Waili NS, El Menyiy N, Imtara H, Figuira AC, Al-Waili T, et al. Antioxidant activity and protective effect of bee bread (honey and pollen) in aluminum-induced anemia, elevation of inflammatory makers and hepato-renal toxicity. *J food sci tech.* (2017) 54:4205-12. doi:10.1007/s13197-017-2889-9

36. Gardana C, Del Bo’ C, Quicazán MC, Corrrea AR, Simonetti P. Nutrients, phytochemicals and botanical origin of commercial bee pollen from different geographical areas. *J Food Compos Anal.* (2018) 73:29-38. doi:10.1016/j.jfca.2018.07.009

37. De-Melo AAM, Estevinho LM, Moreira MM, Delerue-Matos C, Freitas AdSd, Barth OM, et al. A multivariate approach based on physicochemical parameters and biological potential for the botanical and geographical discrimination of Brazilian bee pollen. *Food Biosci.* (2018) 25:91-110. doi:10.1016/j.fbio.2018.08.001

38. Ghosh S, Jung C. Changes in nutritional composition from bee pollen to pollen patty used in bumblebee rearing. *J Asia-Pac Entomol.* (2020) 23:701-8. doi:10.1016/j.aspen.2020.04.008

39. VASILIOS LIOLIOS CT, MARIA DIMOU, DIMITRIOS KANELIS, MARIA-ANNA RODOPOULOU, ANDREAS THRASYVOULOU. Exploring the sugar profile of unifloral bee pollen using high performance liquid chromatography. *J Food Nutr Res-Slov*. (2018) 57:1-10.

40. Özcan MM, Aljuhaimi F, Babiker EE, Uslu N, Ceylan DA, Ghafoor K, et al. Determination of Antioxidant Activity, Phenolic Compound, Mineral Contents and Fatty Acid Compositions of Bee Pollen Grains Collected from Different Locations. [*Journal of Landscape Ecology*](https://webvpn.ujs.edu.cn/http/77726476706e69737468656265737421e3e40f862f3972587b06c7af9758/nav/mag/info?mags=4a720ad418bfcc6a0acbc0f1125ffd30)*.* (2019) 12:26-36. doi: 10.2478/JAS-2019-0004

41. Emmanuel Atsalakisa IC, Maria Makropouloua, Sofia Karabourniotib and Konstantia Graikou. Evaluation of Phenolic Compounds in Cistus creticus Bee Pollen from Greece. Antioxidant and Antimicrobial Properties. *Nat Prod Commun*. (2017) 12:1813-6. doi:10.1177/1934578x1701201141

42. Ozkan K, Sagcan N, Ozulku G, Sagdic O, Toker OS, Muz MN. Bioactive and bioaccessibility characteristics of honeybee pollens collected from different regions of Turkey. *J Food Meas Charact*. (2017) 12:581-7. doi: 10.1007/s11694-017-9670-7

43. Duarte AWF, Vasconcelos MRdS, Oda-Souza M, Oliveira FFd, LÓPez AMQ. Honey and bee pollen produced by Meliponini (Apidae) in Alagoas, Brazil: multivariate analysis of physicochemical and antioxidant profiles. *Food Sci Tech.* (2018) 38:493-503.doi: 10.1590/fst.09317

44. Araujo JS, Chambo ED, Costa M, Cavalcante da Silva SMP, Lopes de Carvalho CA, L ME. Chemical Composition and Biological Activities of Mono- and Heterofloral Bee Pollen of Different Geographical Origins. *Int J mol sci.* (2017) Apr 27;18(5). PubMed PMID: 28448467. Pubmed Central PMCID: 5454834. doi: 10.3390/ijms18050921

45. HernÁNdez Zarate MS, Abraham JuÁRez MdR, CerÓN GarcÍA A, Ozuna LÓPez C, GutiÉRrez ChÁVez AJ, Segoviano Garfias JdJN, et al. Flavonoids, phenolic content, and antioxidant activity of propolis from various areas of Guanajuato, Mexico. *Food Sci Tech.* (2018) 38(2):210-5. doi: 10.1590/fst.29916

46. Thakur M, Nanda V. Screening of Indian bee pollen based on antioxidant properties and polyphenolic composition using UHPLC-DAD-MS/MS: A multivariate analysis and ANN based approach. *Food Res Int*. (2021) 140:110041. doi:10.1016/j.foodres.2020.110041

47. Bujang JS, Zakaria MH, Ramaiya SD. Chemical constituents and phytochemical properties of floral maize pollen. *PloS one*. (2021) 16:e0247327. <https://doi.org/10.1371/journal.pone.0247327>

48. Kurek-Gorecka A, Komosinska-Vassev K, Rzepecka-Stojko A, Olczyk P. Bee Venom in Wound Healing. *Molecules.* (2020) 26:148. Doi:10.3390/molecules26010148

49. Bellik Y. Bee Venom: Its Potential Use in Alternative Medicine. Anti-Infective Agents (2015) 13:3-16. doi:10.2174/2211352513666150318234624

50. Giampieri F, Quiles JL, Orantes-Bermejo FJ, Gasparrini M, Forbes-Hernandez TY, Sanchez-Gonzalez C, et al. Are by-products from beeswax recycling process a new promising source of bioactive compounds with biomedical properties? *Food chem toxicol.* (2018) 112:126-33. doi:10.1016/j.fct.2017.12.041

51. Maghsoudlou A, Mahoonak AS, Mohebodini H, Toldra F. Royal Jelly: Chemistry, Storage and Bioactivities. *J Apic Sci.* (2019) 63:17-40. doi:10.2478/jas-2019-0007

52. Ramanathan ANKG, Nair AJ, Sugunan VS. A review on Royal Jelly proteins and peptides. *J Funct Foods.* (2018) 44:255-64.doi: 10.1016/j.jff.2018.03.008

53. Cornara L, Biagi M, Xiao J, Burlando B. Therapeutic Properties of Bioactive Compounds from Different Honeybee Products. *Front pharmacol*. 2017;8:412. PubMed PMID: 28701955. Pubmed Central PMCID: 5487425.

54. Nagai T, Inoue R. Preparation and the functional properties of water extract and alkaline extract of royal jelly. *Food Chem.* (2004) 84:181-6. doi:10.1016/s0308-8146(03)00198-5

55. Melliou E, Chinou I. Chemistry and Bioactivities of Royal Jelly, Atta-ur- Rahman editor. Studies in Natural Products Chemistry. Elsevier Press. (2014) p.261-90.

56. Ramadan MF, Al-Ghamdi A. Bioactive compounds and health-promoting properties of royal jelly: A review. *J Funct Foods.* (2012) 4(1):39-52. doi:10.3389/fphar.2017.00412

57. Xue X, Wu L, Wang K. "Chemical Composition of Royal Jelly" in José M. Alvarez-Suarez editor. Bee products-Chemical and Biological Properties. Switzerland, Springe press. (2017) 181-190.

58. Kocot J, Kielczykowska M, Luchowska-Kocot D, Kurzepa J, Musik I. Antioxidant Potential of Propolis, Bee Pollen, and Royal Jelly: Possible Medical Application. *Oxid Med Cell Longev.* (2018) 2018: 7074209. doi:10.1155/2018/7074209

59. Kunugi H, Mohammed Ali A. Royal Jelly and Its Components Promote Healthy Aging and Longevity: From Animal Models to Humans. *Int J mol sci*. (2019) 20:4662. doi:10.3390/ijms20194662

60. Kageyama M, Li K, Sun S, Xing G, Gao R, Lei Z, et al. Anti-tumor and anti-metastasis activities of honey bee larvae powder by suppressing the expression of EZH2. *Biomed Pharmacother*. (2018) 105:690-6. doi:10.1016/j.biopha.2018.06.034

61. Kageyama M, Xing G, Li K, Zhang Z, Sugiyama A. Oral administration of freeze-dried powders of honey bee larvae inhibits the development of atopic dermatitis-like skin lesions in NC/Nga mice. *Personalized Medicine Universe*. (2017) 6:22-7. doi:10.1016/j.pmu.2017.05.001

62. Ghosh S, Jung C, Meyer-Rochow VB. Nutritional value and chemical composition of larvae, pupae, and adults of worker honey bee, Apis mellifera ligustica as a sustainable food source. *J Asia-Pac Entomol*. (2016) 19:487-95. doi:10.1016/j.aspen.2016.03.008

63. Isik A, Ozdemir M, Doymaz I. Effect of hot air drying on quality characteristics and physicochemical properties of bee pollen. *Food Sci Tech.* (2019) 39:224-31. doi:10.1590/fst.02818

64. Andrés Duran MCQ, Carlos M. Zuluaga. Effect of Solar Drying Process on Bioactive Compounds and Antioxidant Activity In Vitro of High Andean Region Bee Pollen. *Chem Eng T*. (2019) 75:91-6. doi:10.3303/CET1975016

65. Zuluaga-Domínguez C, Serrato-Bermudez J, Quicazán M. Influence of drying-related operations on microbiological, structural and physicochemical aspects for processing of bee-pollen. *Eng Agri, Environment Food.* (2018) 11(2):57-64. doi: 10.1016/j.eaef.2018.01.003

66. D. E. Kashirin IAU, M. Yu. Kostenko, G. K. Rembalovich, K. I. Danilov, G. D. Kokorev, D. N. Byshov, V. A. Makarov, B. A. Nefedov, A. A. Tsymbal and V. M. Ulyanov Cyclic convective drying of bee pollen. *ARPN J Eng Appl Sci.* (2019) 14:916-20. http://www.arpnjournals.org/jeas/research_papers/rp_2019/jeas_0219_7635.pdf

67. Ranieri A, Benelli G, Castagna A, Sgherri C, Signorini F, Bientinesi M, et al. Freeze-drying duration influences the amino acid and rutin content in honeybee-collected chestnut pollen. *Saudi J biol sci*. (2019) 26:252-5. doi:10.1016/j.sjbs.2017.08.011

68. Dzung NT. Study of determining the technological mode in the freeze drying process of royal jelly in viet nam. *Carpathian J Food Sci.* (2016) 8(2):47-62.

69. MARIA CRISTINA MESSIA MFC, AND EMANUELE MARCONI. Storage Stability Assessment of Freeze-Dried Royal Jelly by Furosine Determination. *J Agric Food Chem*. (2005) 53:4440-3. doi:10.1021/jf0404647

70. Dzung NT, Manh LD, Suc NV. Study Technological Factors Effect on the Loss of Protein, Carbohydrate and Lipid inside Royal Jelly in the Freeze Drying Process. *Current Res J Biol Sci*. (2015) 7(2):22-30. doi:10.19026/crjbs.7.5203

71. Isik A, Ozdemir M, Doymaz I. Infrared drying of bee pollen: effects and impacts on food components. *Czech J Food Sci.* (2019) 37:69-74. doi:10.17221/410/2017-CJFS

72. Conte G, Benelli G, Serra A, Signorini F, Bientinesi M, Nicolella C, et al. Lipid characterization of chestnut and willow honeybee-collected pollen: Impact of freeze-drying and microwave-assisted drying. *J Food Compos Anal.* (2017) 55:12-9. doi: 10.1016/j.jfca.2016.11.001

73. Borel LDMS, Marques LG, Prado MM. Performance evaluation of an infrared heating-assisted fluidized bed dryer for processing bee-pollen grains. *Chem Eng Process.* (2020) 155:108044. doi:10.1016/j.cep.2020.108044

74. Kharchenko S, Oskin S, Tsokur D. Modeling of bee-bread drying process. [*Engineering for Rural Development-International Scientific Conference*](https://webvpn.ujs.edu.cn/http/77726476706e69737468656265737421e3e40f862f3972587b06c7af9758/nav/mag/info?mags=728e84d419b6a980ab857154db6f50c4)*.* (2020) 19:445-49. doi: 10.22616/ERDev.2020.19.TF100

75. Castagna A, Benelli G, Conte G, Sgherri C, Signorini F, Nicolella C, et al. Drying Techniques and Storage: Do They Affect the Nutritional Value of Bee-Collected Pollen? *Molecules*. (2020) 25:E4925. doi:10.3390/molecules25214925

76. Kilic A. Low temperature and high velocity assisted fluidized bed drying characteristics of bee pollen as bioactive food. *J Food Process Eng.* (2020) 43:1-9. doi:10.1111/jfpe.13439

77. Isik A, Ozdemir M, Doymaz I. Investigation of microwave drying on quality attributes, sensory properties and surface structure of bee pollen grains by scanning electron microscopy. *Braz J Chem Eng.* (2021) 38:177-88. doi:10.1007/s43153-020-00088-w

78. Kanar Y, Mazı BG. HMF formation, diastase activity and proline content changes in bee pollen dried by different drying methods. *Lwt*. (2019) 113:108273. doi:10.1016/j.lwt.2019.108273

79. Canale A, Benelli G, Castagna A, Sgherri C, Poli P, Serra A, et al. Microwave-Assisted Drying for the Conservation of Honeybee Pollen. *Materials*. (2016) 9:363. doi:10.3390/ma9050363
